# A solvent-free mechanochemical synthesis of polyaromatic hydrocarbon derivatives†

**DOI:** 10.1039/c9ra04921e

**Published:** 2019-09-04

**Authors:** Cong Wang, Malik Hill, Brandon Theard, James Mack

**Affiliations:** Department of Chemistry, University of Cincinnati 404 Crosley Tower Cincinnati Ohio USA james.mack@uc.edu

## Abstract

Polyaromatic hydrocarbons are central molecules in the future of nanotechnology. However, the synthesis of these molecules is limited by their lack of solubility in solvents, especially green solvents, their ease of oxidation in solution and use of harmful reagents. Solvent-free mechanochemistry has been shown to have excellent potential for these types of molecules and should provide a much more environmentally benign approach for the synthesis of this very important class of molecules. This report details the use of mechanochemistry on an iterative strategy for the synthesis of polyaromatic hydrocarbon derivatives.

## Abstract 2

A solvent-free use of mechanochemistry on an iterative strategy for the synthesis of polyaromatic hydrocarbon derivatives.

## Graphical abstract

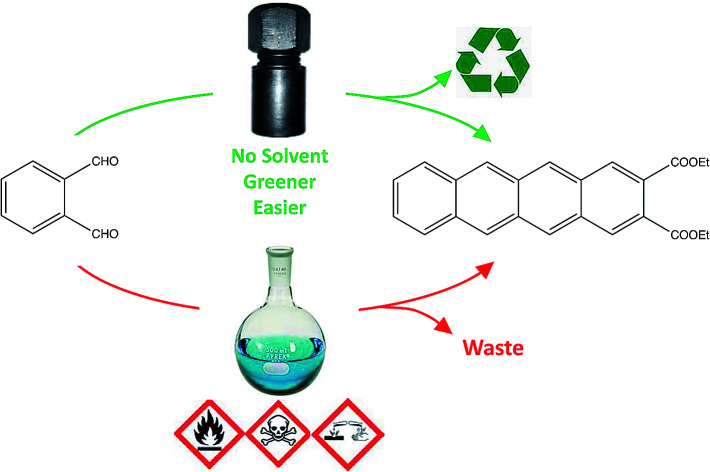

## Introduction

The use of carbon-based molecules is important in the development of nanotechnology. Nanotubes, fullerenes, fullerene fragments and various functionalized polyaromatic hydrocarbons have been at the forefront of the materials landscape.^1–6^ Although these molecules have demonstrated great potential, the synthesis and modification of these molecules are rather challenging. First, given the lack of solubility in a variety of green solvents, solvents such as, methylene chloride, and various other volatile organic compounds are needed in the synthesis of these viable molecules. In addition, various harmful reagents, such as lithium aluminium hydride and butyl lithium are needed to carry out typical organic transformations.^7,8^ Furthermore as linear acenes increase in length, solubility diminishes and these molecules become more susceptible to air oxidation.^9^ In order for this class of molecules to be a viable long-term solution in the field of nanotechnology, we need to develop more sustainable methods to synthesize them, which are safer and significantly reduce the amount of waste generated.

Mechanochemistry could potentially provide a much needed safer and solvent-free alternative. Over the years mechanochemistry has been demonstrated to provide many chemical transformations in a solvent-free environment.^10–18^ Oxidations,^19,20^ reductions,^21,22^ and coupling reactions^23,24^ have all been performed in high yields under these unique conditions. Herein, we describe a mechanochemical synthesis of various linear acenes.

In addition to the sustainability advantages, we believe mechanochemistry would have additional advantages for the synthesis of polyaromatic hydrocarbons. While some linear acenes are soluble in common organic solvents, most are not. According to the CRC Handbook of Chemistry and Physics, naphthalene shows solubility in green solvents like ethanol and acetone, while pentacene exhibits no solubility in ethanol or acetone and shows only slight solubility in benzene and nitrobenzene. Therefore, as the number of rings are increased, solubility is significantly decreased, making it more difficult to synthesize these molecules in solution, especially in a green solvent.^9^ Mechanochemistry has been shown to alleviate this problem with fullerenes and nanotubes, whose solubility in various organic solvents is minimal, but modifications by mechanochemistry are excellent.^25–27^ Another potential benefit of the mechanochemical synthesis of polyaromatic hydrocarbons under solvent-free conditions is the increased stability these molecules exhibit in the solid state. Although these molecules are stable in the solid state under aerobic conditions, they are easily oxidized in solution to the corresponding quinone, whereas the longer the chain, the easier it is to oxidize.

## Results and discussion

**Scheme 1:**
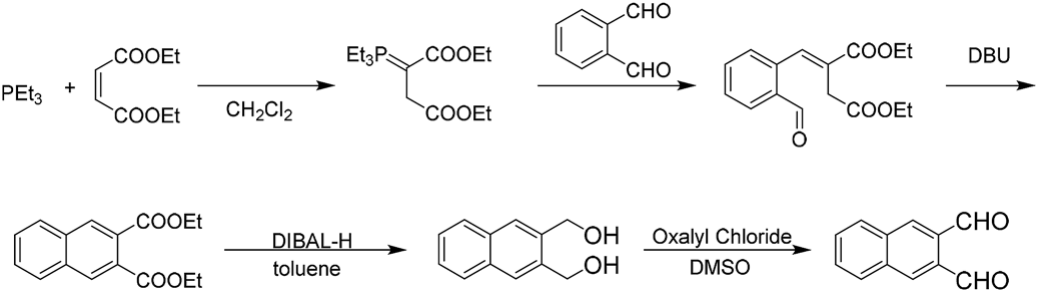
Solution based iterative synthesis to extend ring expansion from phthalaldehyde to naphthalene-2,3 dicarboxaldehyde.

To begin to understand the benefit of mechanochemistry to the synthesis of polyaromatic hydrocarbons we explored the successive polyaromatic hydrocarbon synthesis of Lin and co-workers.^28^ Through successive reactions such as the Wittig reaction, base catalysed ring closure, hydride reduction, followed by Swern oxidation, Lin demonstrated the ability to additionally add rings starting with a naphthalene core (Scheme 1).

Although this synthesis demonstrated a novel approach to the synthesis of linear polyaromatic hydrocarbons, from a green perspective there were various points in the synthesis that were not sustainable. Our goal was to examine each step in the synthesis and provide an appropriate green alternative that would make the synthesis of these important molecules more sustainable.

**Scheme 2:**
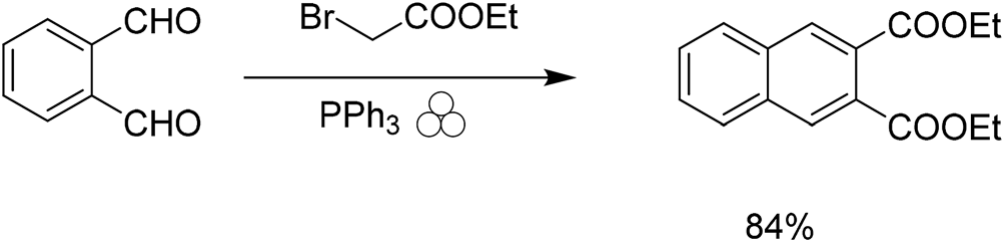
Mechanochemical synthesis of naphthalene-2,3-dicarboxylic acid diethyl ester.

The first step of the reaction calls for the use of triethyl phosphine, a highly flammable compound with a strong odor. We wanted to find a more sustainable replacement for the use of triethyl phosphine in the Wittig reaction. Given the previous success of the Wittig reaction under mechanochemical conditions,^29–31^ we attempted to use the safer and more versatile triphenyl phosphine as a replacement; however, this simple replacement gave poor yields. Siebum and co workers demonstrated the use of triphenylphosphine and bromo ethylacetate to give the corresponding ylide in 75% yield in solution.^32^ We attempted this reaction under solvent-free conditions to determine if we could produce naphthalene-2,3-dicarboxylic acid diethyl ester from 1,2-phthalic dicarboxaldehyde. Initially the reaction gave low yields of products using one stainless steel ball, however when we used multiple milling balls the reaction gave the desired diester naphthalene in 84% yield (Scheme 2).

Lin's ring closure step required 1,8-diazabicyclo[5.4.0]undec-7-ene (DBU), which can be corrosive. Following the ring closure, they performed a DiBAL-H reduction in toluene, followed by a subsequent Swern oxidation to give the desired aldehyde. DiBAL-H is extremely hazardous and Swern uses oxalyl chloride which has a number of hazards associated with it. In addition to the use of harmful reagents, solvents such as methylene chloride, tetrahydrofuran and dimethyl sulfoxide were needed and most of the reactions were performed under a nitrogen environment at very low temperatures.

**Scheme 3:**
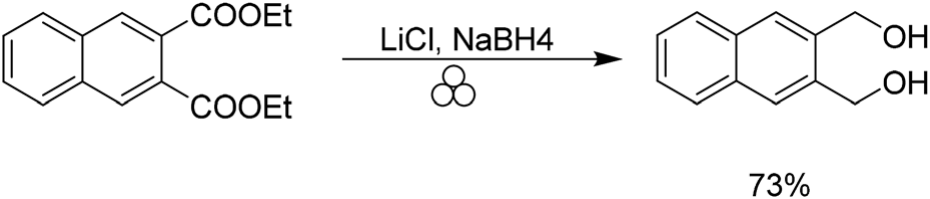
Mechanochemical synthesis of 2,3 bis(hydroxymethyl)naphthalene.

Our group has previously demonstrated the ability to use a combination of lithium chloride in the presence of sodium borohydride to reduce esters in a safe solvent-free manner.^33^ Typically, the ester functional group is reduced in the presence of lithium aluminium hydride or diisobutyl aluminium hydride at low temperatures. We were able to reduce the aforementioned 1,2-phthalic dicarboxaldehyde in 73% yield to the resulting diol (Scheme 3).

**Scheme 4:**
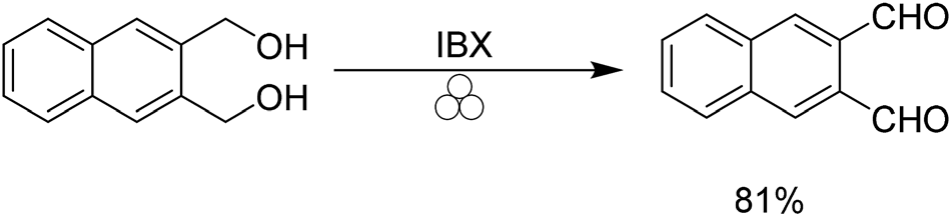
Mechanochemical oxidation using IBX.

Recently Achar and co-workers demonstrated the ability to oxidize alcohols to their corresponding aldehydes using IBX under mechanochemical conditions (Scheme 4).^34^ We were able to apply these conditions to 2,3 bis(hydroxymethyl)naphthalene to provide naphthalene-2,3 dicarboxaldehyde in 81% yield.

**Scheme 5:**
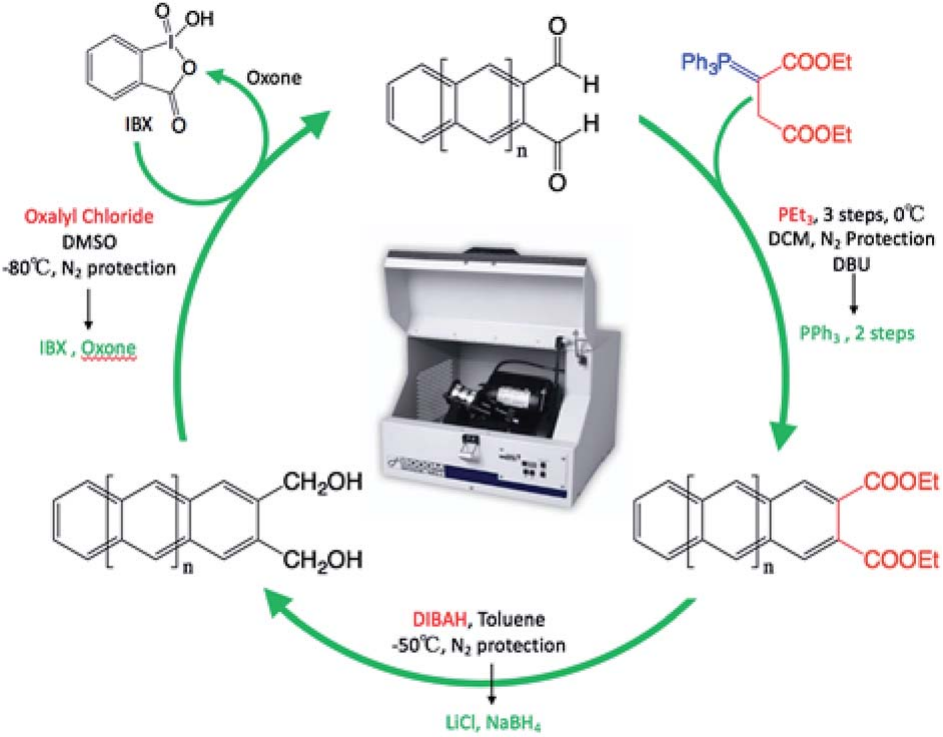
Repeatable synthetic cycle for the synthesis of linear acenes under mechanochemical conditions. (Red font is traditional solutions based, where green font is solvent-free conditions.)

Once we obtained naphthalene-2,3 dicarboxaldehyde, we were able to repeat the cycle to obtain the anthracene-2,3 dicarboxaldehyde and tetracene-2,3 dicarboxaldehyde (Scheme 5).

**Scheme 6:**
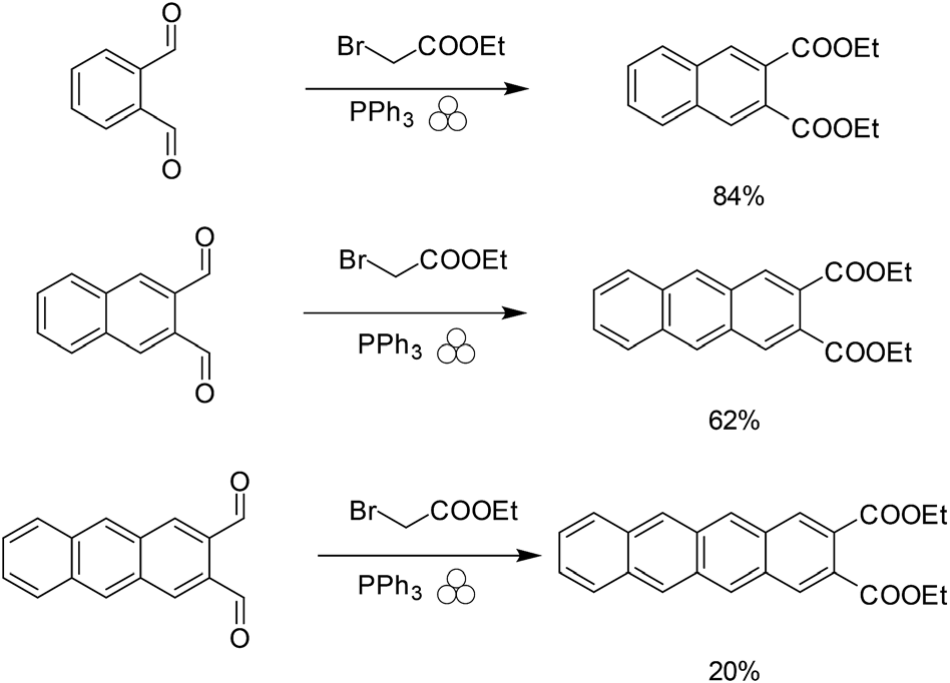
Difference in rate between naphthalene derivatives and anthracene derivatives.

Although we were able to obtain anthracene-2,3 dicarboxaldehyde and tetracene-2,3 dicarboxaldehyde we observed rate differences between the naphthalene derivative and the anthracene and tetracene derivatives (Scheme 6).

We noticed that each step of the anthracene substrate was slower than the naphthalene substrate although the functional groups of the reaction and reagents were the same. Our initial thought was that this was related to the rheology of the materials, as have been observed in other mechanochemical processes.^35,36^ In order to gain a better understanding of this phenomenon, we added milling aids such as silica gel, which has been shown to provide significant rate enhancements of mechanochemical processes.^37–40^ Upon adding silica gel to the reaction mixture, we observed a significant rate enhancement on the reactivity of the anthracene substrates. Currently, we are investigating the influence of silica gel on these reactions in order to get a better understanding of this effect.

We wanted to better quantify the sustainability of our process using the Ecoscale.^41^ Each step of our solvent-free synthesis was more sustainable than the solution synthetic step (see ESI†). Additionally, each step was calculated to be either acceptable or excellent according to the Ecoscale metric. Furthermore, our synthesis was roughly 3–4 times less expensive to synthesize than the tradition solution-based route. Demonstrating the power of the synthesis under mechanochemical solvent-free methodology.

## Conclusion

To conclude we have demonstrated a solvent-free mechanochemical synthetic pathway for the synthesis of polyaromatic hydrocarbons. Each step was demonstrated to be more environmentally benign according to the Eco scale. We demonstrated that similar to solution-based conditions this iterative process can be extended to incorporate long acene formation.

## Conflicts of interest

There are no conflicts to declare.

## Supplementary Material

RA-009-C9RA04921E-s001
