# Supplementary material for: A solvent-free mechanochemical synthesis of polyaromatic hydrocarbon derivatives
Source: RSC Adv. 2019 Sep 4;9(48):27888–91. doi: 10.1039/c9ra04921e (PMC9070754; doi:10.1039/c9ra04921e)
Supplement: RA-009-C9RA04921E-s001 [file RA-009-C9RA04921E-s001.pdf]

Electronic Supplementary Material (ESI) for RSC Advances.
This journal is © The Royal Society of Chemistry 2019

A solvent-free mechanochemical synthesis of polyaromatic hydrocarbon derivatives

Cong Wang, Malik Hill, James Mack

Department of Chemistry, University of Cincinnati, 301 Clifton Court, Cincinnati, Ohio 45221-0172, United states

Supporting Information

Table of contents

General information……………………………………………………………………………………S2
Experimental………………………………………………………………………………………………S2
Optimization study………………………………………………………………………………………S5
Spectra………………………………………………………………………………………………………S8
Eco Scale Calculation…………………………………………………………………………………..S29
Reference…………………………………………………………………………………………………S43

General information

All NMR spectra were recorded on a Bruker Avance 400 spectrometer. Deuterated NMR solvents were obtained from Cambridge Isotope Laboratories, Inc., and used without further purification. For high resolution MS data, sample was analyzed by using an Orbitrap Fusion Lumos Mass Spectrometer for high resolution data. GC-MS data was obtained from Agilent Technologies 7890B GC system and 5977A MSD. All chemicals were purchased from Acros Organics and used without further purification. Mechanochemistry was carried out in an 8000 M Spex Certiprep Mixer/Mill. And all the reactions were conducted at 18Hz. Reaction vials (Smartsnap grinding jar) were purchased from Form-Tech Scientific. Ball bearings were purchased from Small Parts incorporated. ~1g for one 1/4" stainless steel ball (S.S. ball). Flash column system (CombiFlash Rf+) was purchased from Teledyne ISCO.

Experimental

Naphthalene-2,3-dicarboxylic acid diethyl ester (CAS No. 50919-54-5)

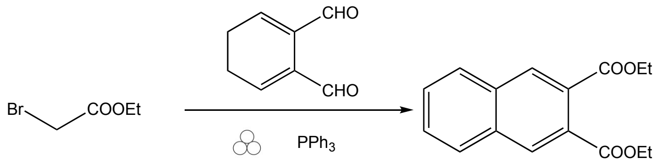

1.57g (6mmol, 1.2eq.) PPh_3 2.17g (13mmol, 2.6eq.) ethyl bromoacetate 3.6g (26mmol, 5.2eq.) K_2CO_3 was put in a 30ml milling vial with 25 1/4" S.S. balls (total weight is 26.748g). The vial was placed in an 8000 M Spex Certiprep mixer/mill and the contents were ball milled for 4h. Wait until vial get cool down to room temperature. Open the vial carefully. 0.56g (5mmol, 1eq.) 1,2-Phthalic dicarboxaldehyde was put into the vial. The vial was placed in the mill and the contents were ball milled for 16h, 18Hz. After it is done, 3 portions ethyl acetate (10ml for each portion) was used to remove the mixture from the vial. Then, gravity filtration was used to remove the solid. Solution was collected and silica gel was added and solvent was removed under reduced pressure. The resulting mixture was purified by flash column chromatography on silica gel (EtOAc:Heptane=1:9). White to pale yellow solid was obtained. Yield 84%.

^{1}H NMR ($CDCl_3$, 400 MHz, ppm): δ 1.39 (t, 6H), 4.41 (q, 4H), 7.56-7.59 (m, 2H), 7.87-7.89 (m, 2H), 8.23 (s, 2H) 2H); ^{13}C NMR ($CDCl_3$, 400 MHz, ppm): δ 14.19, 61.64, 128.45, 128.59, 128.84, 129.99, 133.33, 167.73; High Resolution MS (HRMS, ESI-MS). (M+H$^+$), ($C_{16}H_{16}O_4$)H$^+$, Calc: 273.1121; Found: 273.1120. Spectrum data match with previous report.[1] Melting point 54-55℃(lit.[2] mp 54-55℃).

(3-Hydroxymethyl-naphthalen-2-yl)-methanol (CAS No. 31554-15-1)

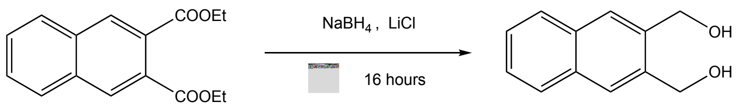

0.81g (3mmol, 1eq.) Naphthalene-2,3-dicarboxylic acid diethyl ester, 0.45g (12mmol, 4eq.) $NaBH_4$, 1.3g (30mmol, 10eq.) LiCl was put in a 15ml milling jar with 7 1/4" stainless balls. The vial was placed in an 8000 M Spex Certiprep mixer/mill and the contents were ball milled for 16h. The mixture was removed into a 250ml beaker and quenched with ~10% HCl (~75ml) and extracted with 3 portions of ethyl acetate (50ml for each portion). The resulting solution was dried with $MgSO_4$ and solvent was removed under reduced pressure and further purified by flash column chromatography on silica gel (EtOAc:Heptane=8:2). White solid was obtained. Yield 75%.

^1H NMR (Acetone-d_6, 400 MHz, ppm): δ 4.87 (d, 4H), 7.47-7.49 (m, 2H), 7.87-7.89 (m, 2H), 7.91 (s, 2H); ^{13}C NMR (Acetone-d_6, 400 MHz, ppm): δ 63.05, 126.59, 127.19, 128.34, 133.77, 139.13; HRMS. (M+Na^+), ($C_{12}H_{12}O_2$)Na^+, Calc: 211.0730; Found: 211.0731. Spectrum data match with previous report.[1] Melting point 157-162℃(lit.[3] mp 155-160℃).

Naphthalene-2,3-dicarbaldehyde (CAS No. 7149-49-7)

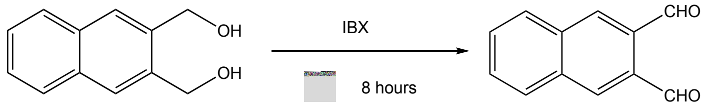

0.33g (1.8mmol, 1eq.) (3-Hydroxymethyl-naphthalen-2-yl)-methanol, 1.5g (5.4mmol, 3eq.) IBX (2-Iodoxybenzoic acid, prepared and recycled in our lab by following the previously reported protocol[4]) was put in a 15ml milling jar with 3 1/4" stainless balls. The vial was placed in an 8000 M Spex Certiprep mixer/mill and the contents were ball milled for 8h. EtOAc (3 portions, 5ml for each portion) was added into the vial. Gravity filtration was used to remove the solid and solution was collected and further purified by flash column chromatography on silica gel (EtOAc:Heptane=4:6). White solid was obtained. Yield 81%.

^1H NMR ($CDCl_3$, 400 MHz, ppm): δ 7.72-7.74 (m, 2H), 8.02-8.05 (m, 2H), 8.42 (s, 2H), 10.61 (s, 2H). ^{13}C NMR ($CDCl_3$, 400 MHz,ppm): δ 129.66, 130.03, 132.84, 134.44, 134.49, 192.52. Spectrum data match with previous report.[1] GC-MS, m/z, 184, [M^+]. Melting point 128-129℃(lit.[5] mp 112-115℃).

Anthracene-2,3-dicarboxylic acid diethyl ester (CAS No. 84041-04-3)

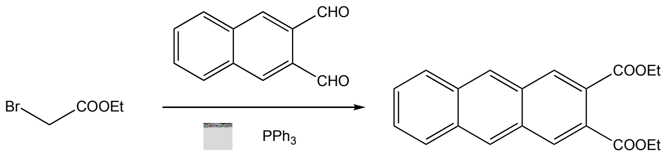

0.78g (3mmol, 1.2eq.) PPh_3 1.1g (6.5mmol, 2.6eq.) ethyl bromoacetate 1.8g (13mmol, 5.2eq.)

K_2CO_3 was put in a 30ml milling jar with 25 1/4" stainless balls. The vial was placed in an 8000 M Spex Certiprep mixer/mill and the contents were ball milled for 4h. Wait until vial get cool down to room temperature. Open the vial carefully. 0.46g (2.5mmol, 1eq.) Naphthalene-2,3-dicarbaldehyde was put into the vial. The vial was placed in the mill and the contents were ball milled for 16h. After it is done, ethyl acetate (3 portions, 10ml for each portion) was used to remove the mixture from the vial. Then gravity filtration was used to remove the solid. Solution was collected and Si gel was added and solvent was removed under reduced pressure. The resulting mixture was purified by flash column chromatography on silica gel (Acetone:Heptane=1:9). Bright yellow solid was obtained. Yield 62%.

^1H NMR ($CDCl_3$, 400 MHz, ppm): δ 1.43 (t, 6H), 4.44 (q, 4H), 7.55-7.57 (m,2H), 8.04-8.06 (m, 2H), 8.44 (s, 2H), 8.52 (s, 2H). ^{13}C NMR ($CDCl_3$, 400 MHz,ppm): δ 14.25, 61.66, 126.79, 127.94, 128.00, 138.42, 130.49, 131.14, 133.06, 167.78. Spectrum data match with previous report.[1] GC-MS, m/z, 322, [M^+]. Melting point 86-87℃(lit.[2] mp 99-100℃).

(3-Hydroxymethyl-anthracen-2-yl)-methanol (CAS No. 1134642-63-9)

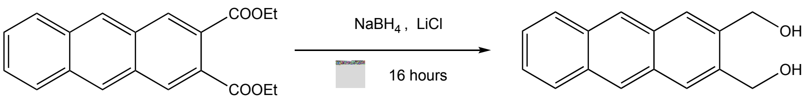

0.322g (1mmol, 1eq.) Anthracene-2,3-dicarboxylic acid diethyl ester, 0.15g (4mmol, 4eq.) $NaBH_4$, 0.424g (10mmol, 10eq.) LiCl was put in a 15ml milling jar with 7 1/4" stainless balls. The vial was placed in an 8000 M Spex Certiprep mixer/mill and the contents were ball milled for 16h. The mixture was removed into a 250ml beaker and quenched with ~10% HCl (~75ml). Vacuum filtration was used and the solid was washed with acetone 3 times (5ml each time). Keep the vacuum on until it is dry. Gray solid was obtained. Yield 55%.

^1H NMR (DMSO-d_6, 400 MHz, ppm): δ 4.74-4.75 (d, 4H), 5.31-5.33 (t, 2H), 7.48-7.50 (m, 2H), 8.04 (s, 2H), 8.06-8.08 (m, 2H), 8.53 (s, 2H) ^{13}C NMR (DMSO-d_6, 400 MHz,ppm): δ 60.68, 124.56, 125.14, 125.29, 127.90, 130.48, 131.07, 138.16. Spectrum data match with previous report.[1] HRMS, ($M+Na^+$), $(C_{16}H_{14}O_2)Na^+$ Calc: 261.0886; Found: 261.0887. Melting point 234-245℃.

Anhracene-2,3-dicarbaldehyde (CAS No.76197-35-8)

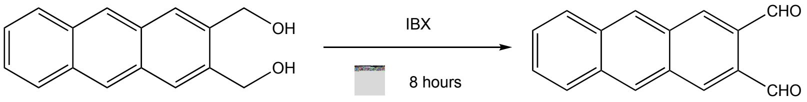

0.23g (1mmol, 1eq.) (3-Hydroxymethyl-anthracen-2-yl)-methanol, 0.84g (3mmol, 3eq.) IBX and ~0.5g silica gel was put in a 15ml milling jar with 3 1/4" stainless balls. The vial was placed in an 8000 M Spex Certiprep mixer/mill and the contents were ball milled for 8h. Acetone (3 portions, 20ml for each portion) was used to remove the mixture form vial. Then gravity

filtration was used to remove solid. Filtrate was collected and dried under reduced pressure. The solid was heated in sublime apparatus at 100°C. The dark orange solid left on the bottom in sublime apparatus was collected. Yield 81%.

^{1}H NMR ($CDCl_3$,400 MHz, ppm): δ 7.64-7.66 (m, 2H), 8.10-8.12 (m, 2H), 8.63 (s, 2H), 8.65 (s, 2H), 10.66 (s, 2H). ^{13}C NMR ($CDCl_3$, 400 MHz,ppm): δ 127.79, 128.67, 129.58, 130.93, 132.04, 133.82, 136.48, 192.48. GCMS Spectrum data match with previous report.[1] GC-MS, m/z, 234, [M^+]. Melting point 197-201℃(lit.[6] mp 217℃).

Naphthacene-2,3-dicarboxylic acid diethyl ester

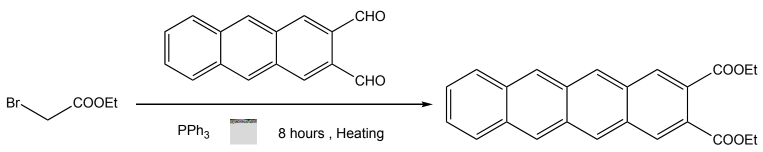

0.157g (0.6mmol, 1.2eq.) PPh_3 0.212g (1.3mmol, 2.6eq.) ethyl bromoacetate 0.36g (2.6mmol, 5.2eq.)K_2CO_3 was put in a 15ml milling jar with 10 1/4" stainless balls. The vial was placed in an 8000 M Spex Certiprep mixer/mill and the contents were ball milled for 4h. Wait until vial get cool down to room temperature. Open the vial carefully. 0.117g (0.5mmol, 1eq.) Anhracene-2,3-dicarbaldehyde was put into the vial. The vial was placed in the mill with the heating apparatus that reported previously in our group[7] and the contents were ball milled and heated at 80°C for 8h. After it is done, ethyl acetate (3 portions, 10ml for each portion) was used to remove the mixture from the vial. Gravity filtration was used to remove the solid. Solution was collected and silica gel was added and solvent was removed under reduced pressure. The resulting mixture was purified by flash column chromatography on silica gel (Acetone:Heptane=1:9). Bright orange solid was obtained. Yield 20%.

^{1}H NMR ($CDCl_3$, 400 MHz, ppm): δ 1.44 (t, J = 7.1 Hz, 6H), 4.44 (q, J = 7.2 Hz, 4H), 7.45-7.48 (m, 2H), 8.02-8.04 (m, 2H), 8.43 (s, 2H), 8.71 (s, 2H), 8.75 (s,2H). ^{13}C NMR ($CDCl_3$, 400 MHz,ppm): δ 14.26, 61.62, 126.03, 126.99, 127.66, 128.34, 128.56, 129.86, 131.02, 131.68, 132.27, 167.72. Spectrum data match with previous report.[1] HRMS. (M+Na^+), ($C_{24}H_{20}O_4$)Na^+, Calc: 395.12538; Found: 395.12541. Melting point 183-186℃.

Optimization study

Control reaction was conducted to find the condition for wittig reaction. Different Stainless steel (S.S.) ball and time length were used.

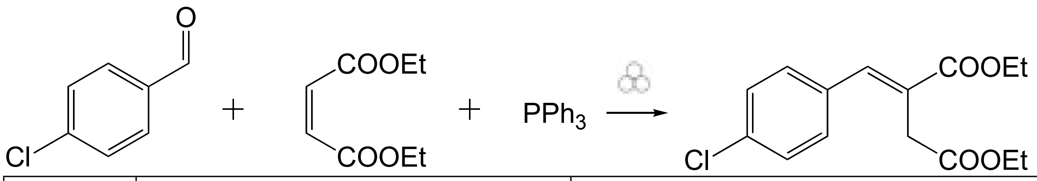

Trial No.	Condition	Yield of diethyl (E)-2-(4-chlorobenzylidene)succinate %

1	1 h, one 3/16-inch SS ball	4
2	16 h, one 3/16-inch SS ball	21
3	1 h, three 3/16-inch SS ball	7
4	16 h, one 3/16-inch SS ball, LiBr	7
5	16 h, nine 3/16-inch SS ball, LiBr	12

Different nucleophilic reagent was used to see if it works better with maleic anhydride. (:Nu = nucleophilic reagent)

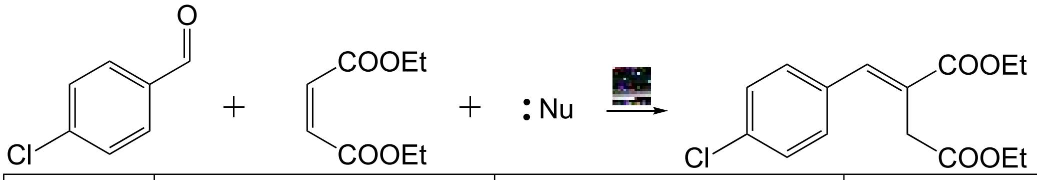

Trial No.	Condition	:Nu	Result
1	16 h, one 3/16-inch SS bal	Tricyclohexylphosphine	No reaction
2	16 h, one 3/16-inch SS bal	DABCO	No reaction
3	16 h, one 3/16-inch SS bal	NaH	inseparable multiple product

Optimization for wittig reaction.

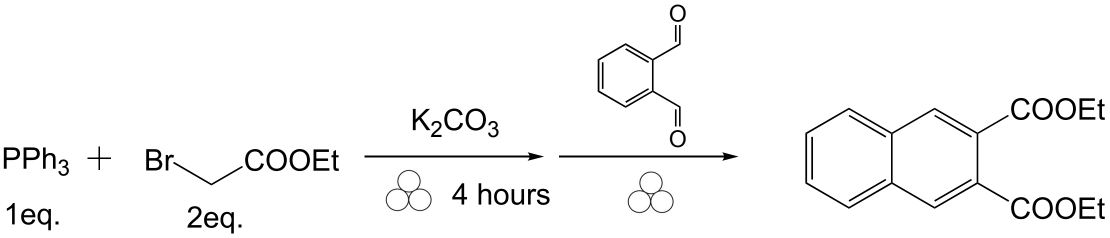

Trial No.	Condition	Yield %
1	one 3/16-inch S.S. balls, 4hours, 15ml milling vial	19
2	one 1/4-inch S.S. balls, 4hours, 15ml milling vial	29
3	Fifteen 3/16-inch S.S. balls, 4hours, 15ml milling vial	51
4	25 1/4-inch S.S. balls, 16hours, 30ml milling vial	84

Optimization for IBX oxidation generating Anhracene-2,3-dicarbaldehyde

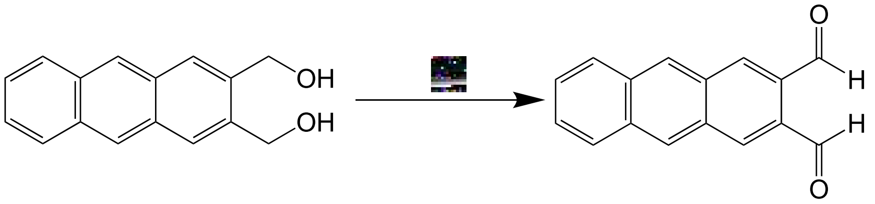

Trial No.	Condition	Yield %
1	3eq. IBX	8
2	6eq. IBX	11
3	3eq. IBX , 1ml EtOAc	8
4	3eq. IBX , Heating(~100℃)	10

5	2.6eq. Dess-Martin reagent	13
6	3eq. IBX , 0.5g Silica gel	81

Optimization for wittig reaction generating Naphthacene-2,3-dicarboxylic acid diethyl ester

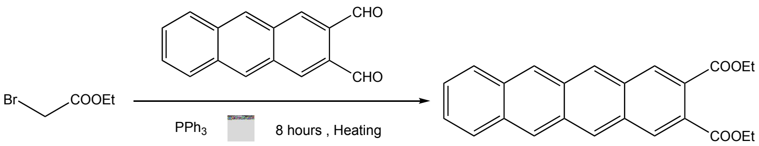

Trial No.	Condition	Yield
1	25 1/4" S.S. balls, 16h	13%
2	25 1/4" S.S. balls, 16h, Ball mill with cooling fan	0%
3	25 1/4" S.S. balls, 16h, ~0.5g dry silica gel	8%
4	25 1/4" S.S. balls, 8h, heating ~80℃	20%

No optimization for other steps.

^{1}H-NMR of Naphthalene-2,3-dicarboxylic acid diethyl ester

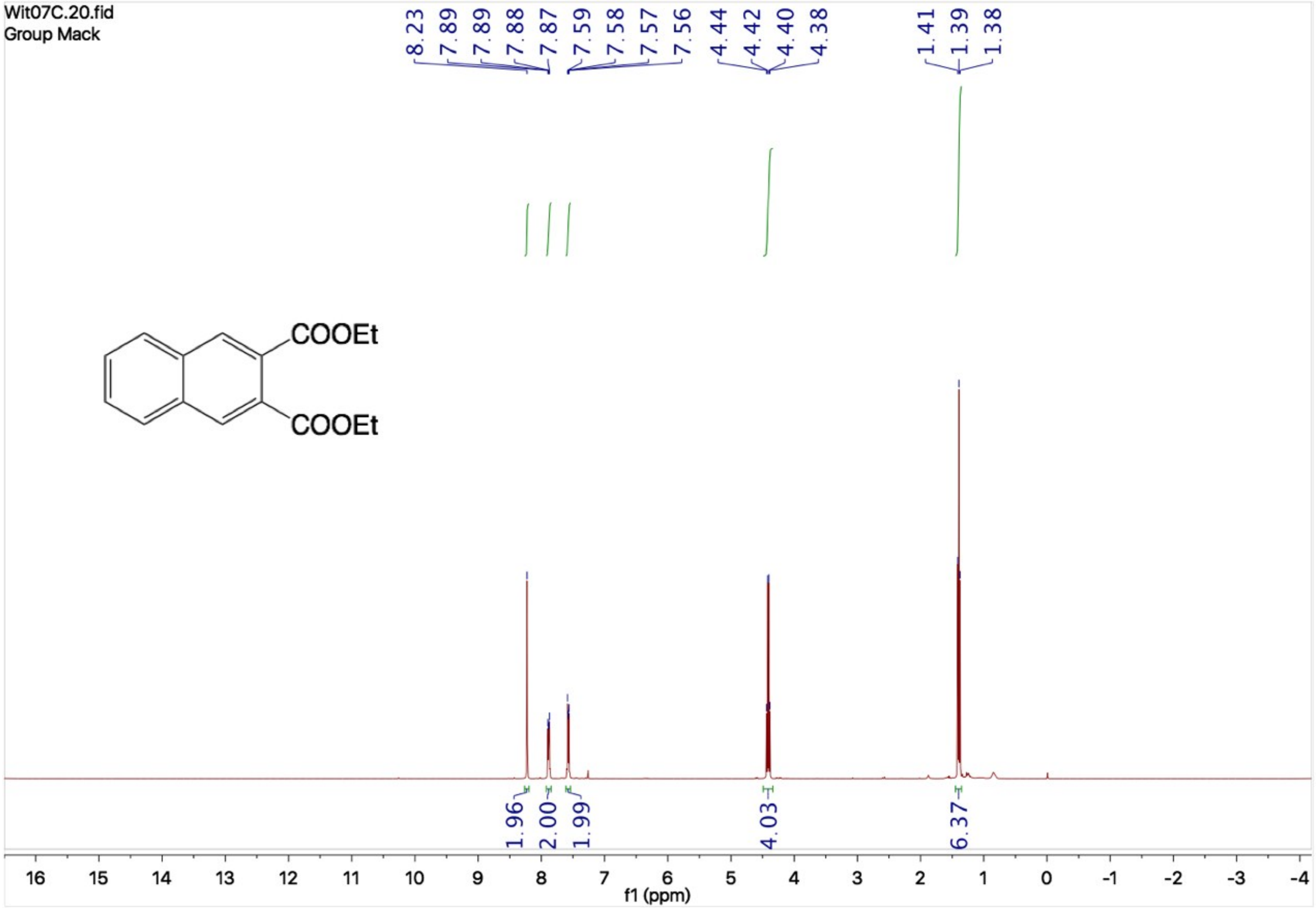

^{13}C-NMR of Naphthalene-2,3-dicarboxylic acid diethyl ester

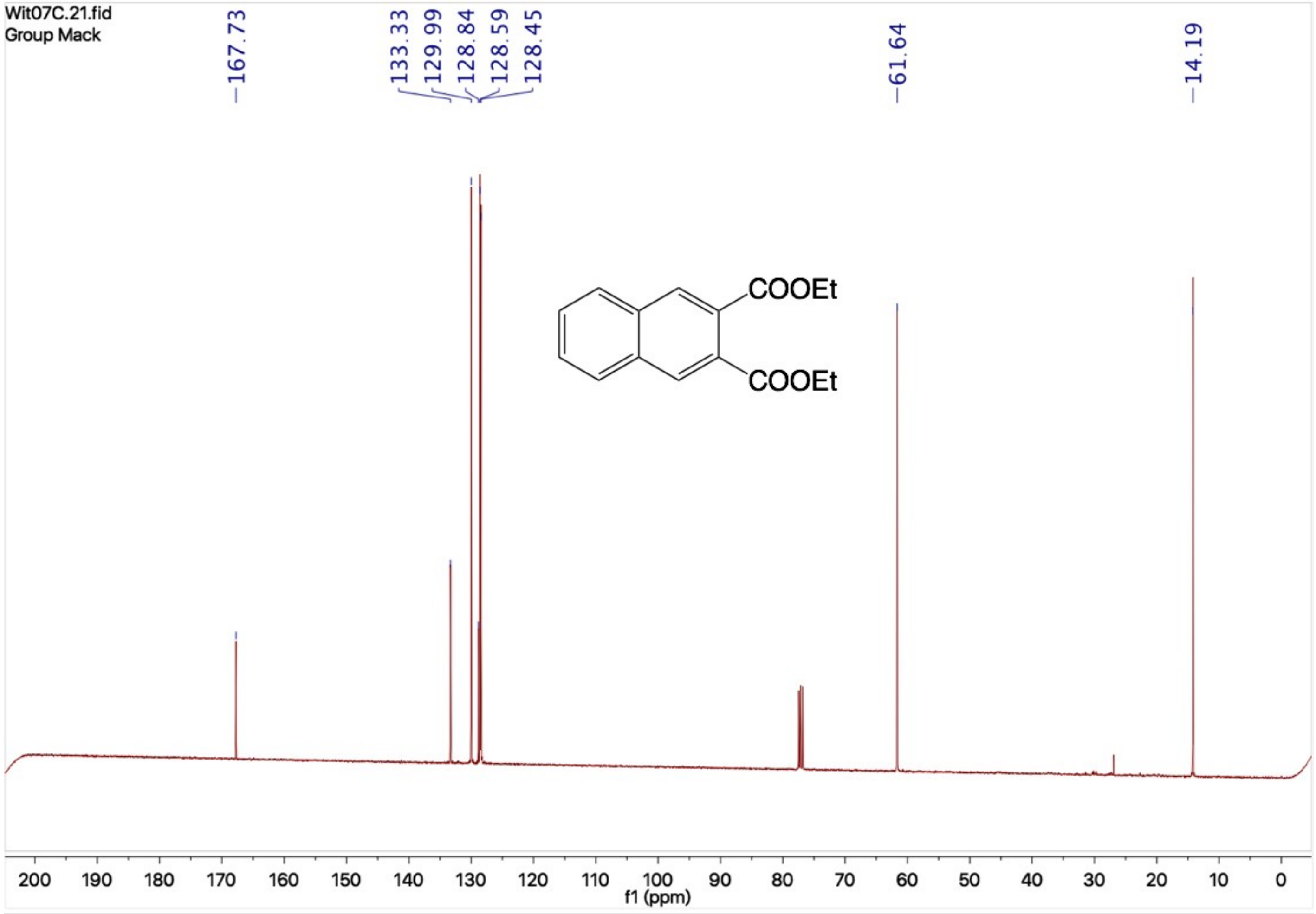

HRMS of Naphthalene-2,3-dicarboxylic acid diethyl ester, (M+H$^+$), $(C_{16}H_{16}O_4)H^+$

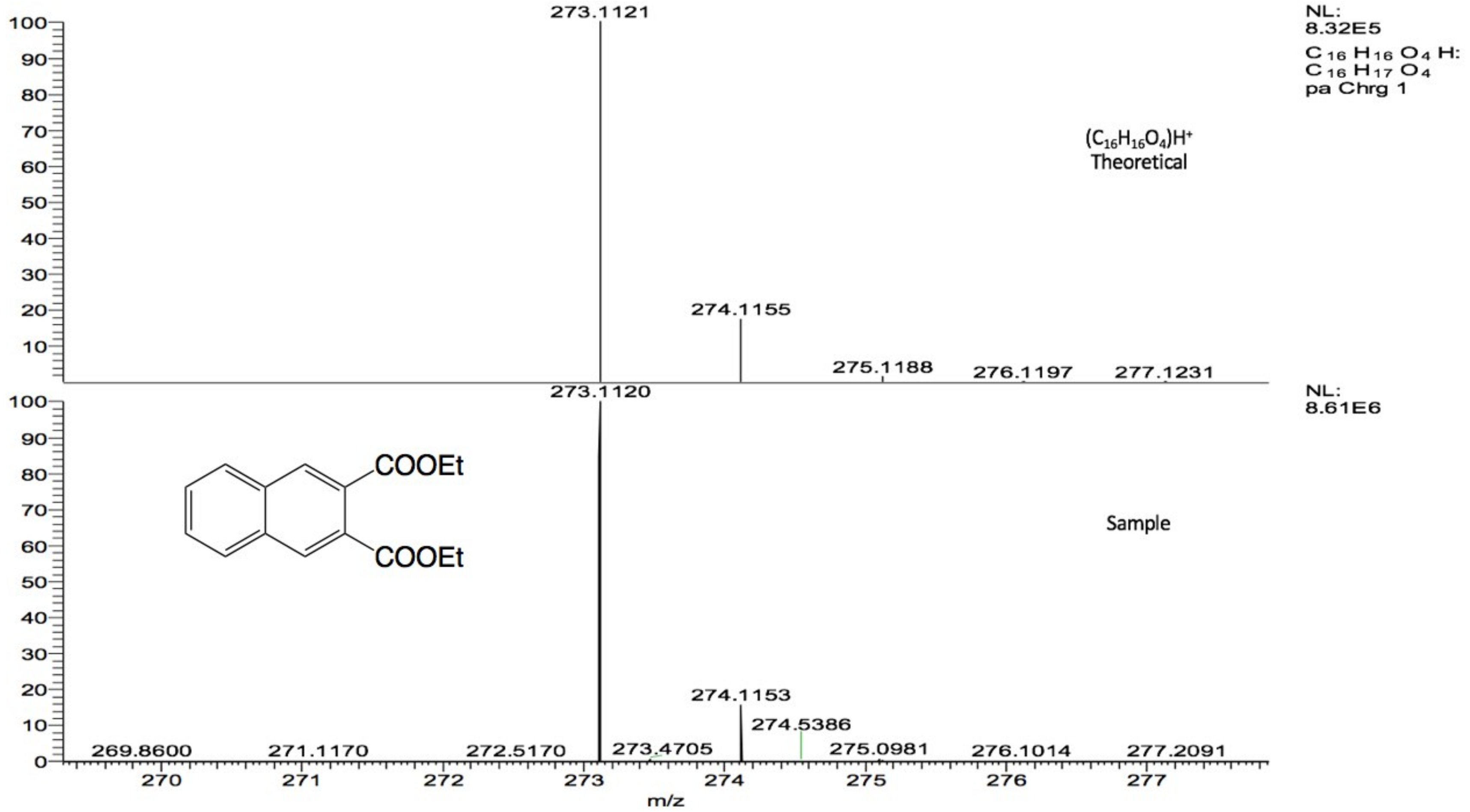

^{1}H-NMR of (3-Hydroxymethyl-naphthalen-2-yl)-methanol

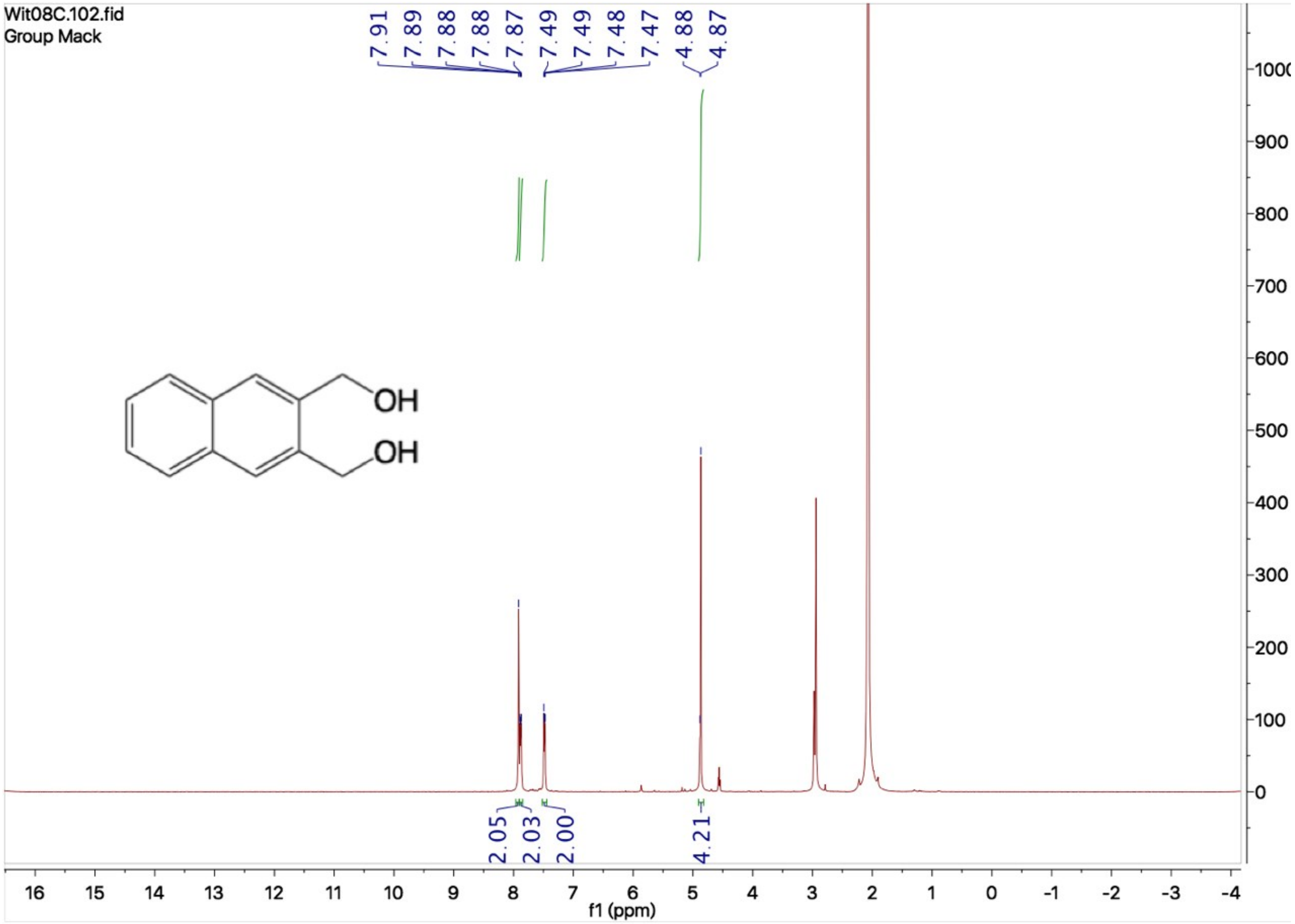

^{13}C-NMR of (3-Hydroxymethyl-naphthalen-2-yl)-methanol

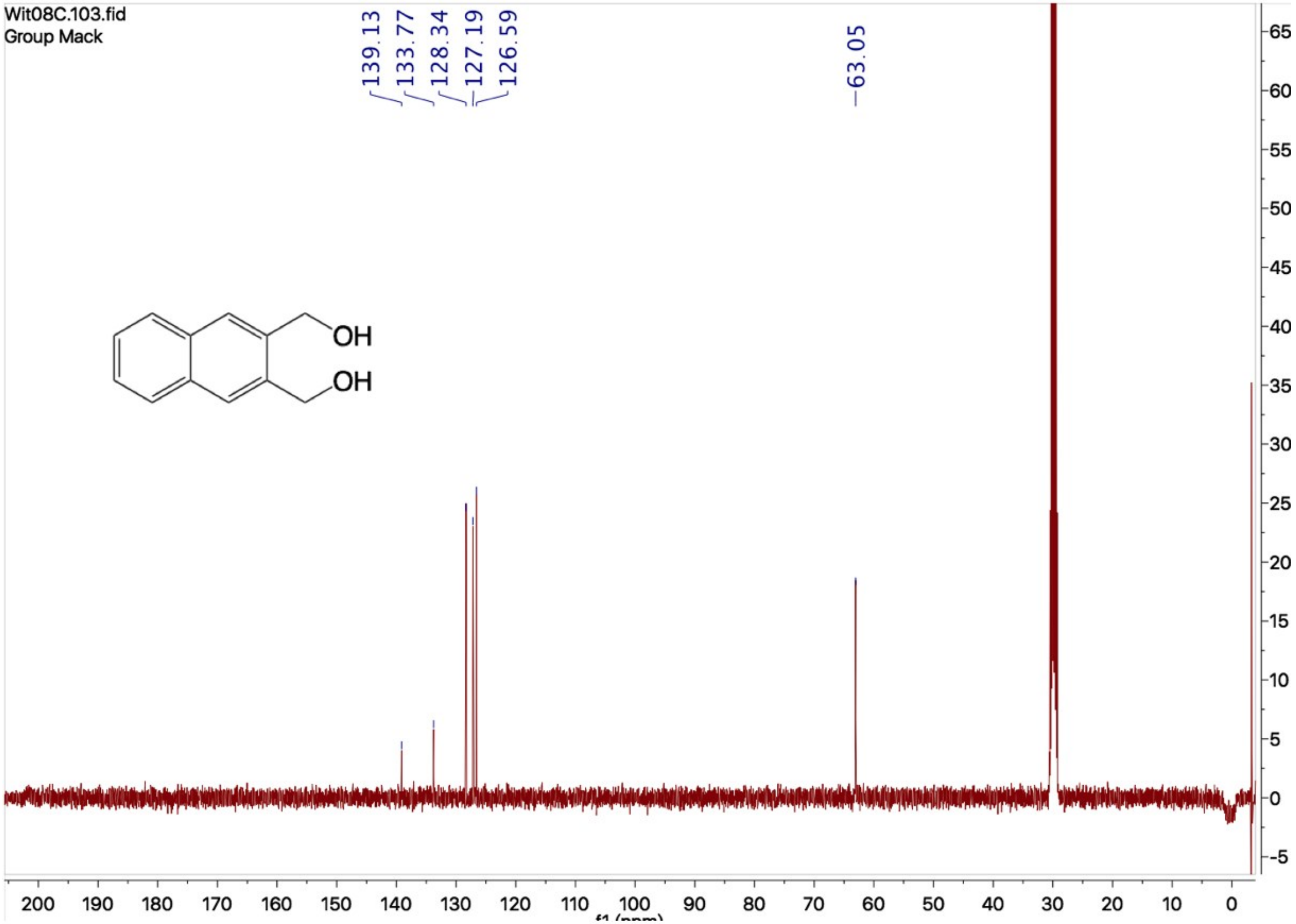

HRMS of (3-Hydroxymethyl-naphthalen-2-yl)-methanol, (M+Na$^+$), $(C_{12}H_{12}O_2)Na^+$

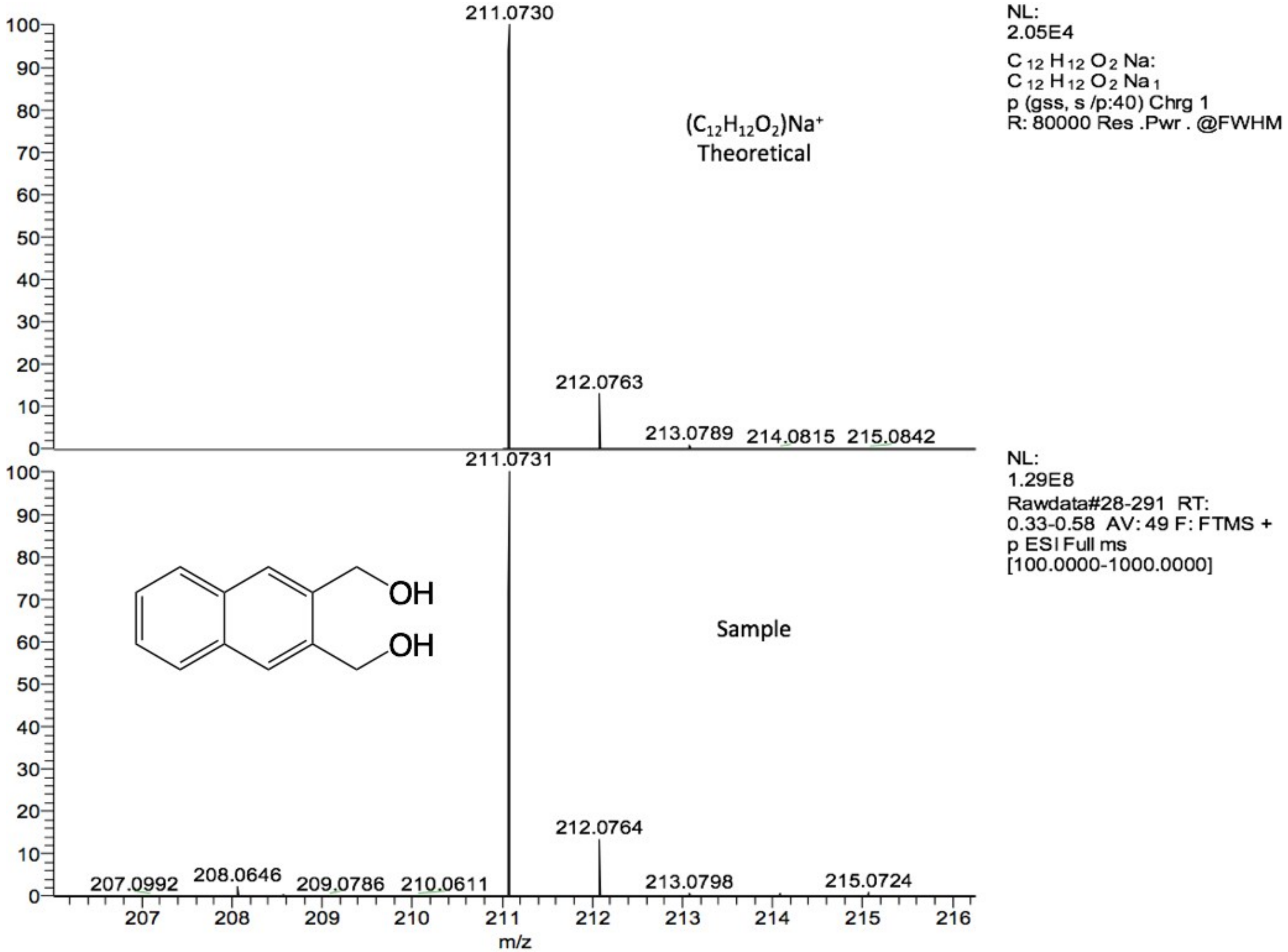

^{1}H-NMR of Naphthalene-2,3-dicarbaldehyde

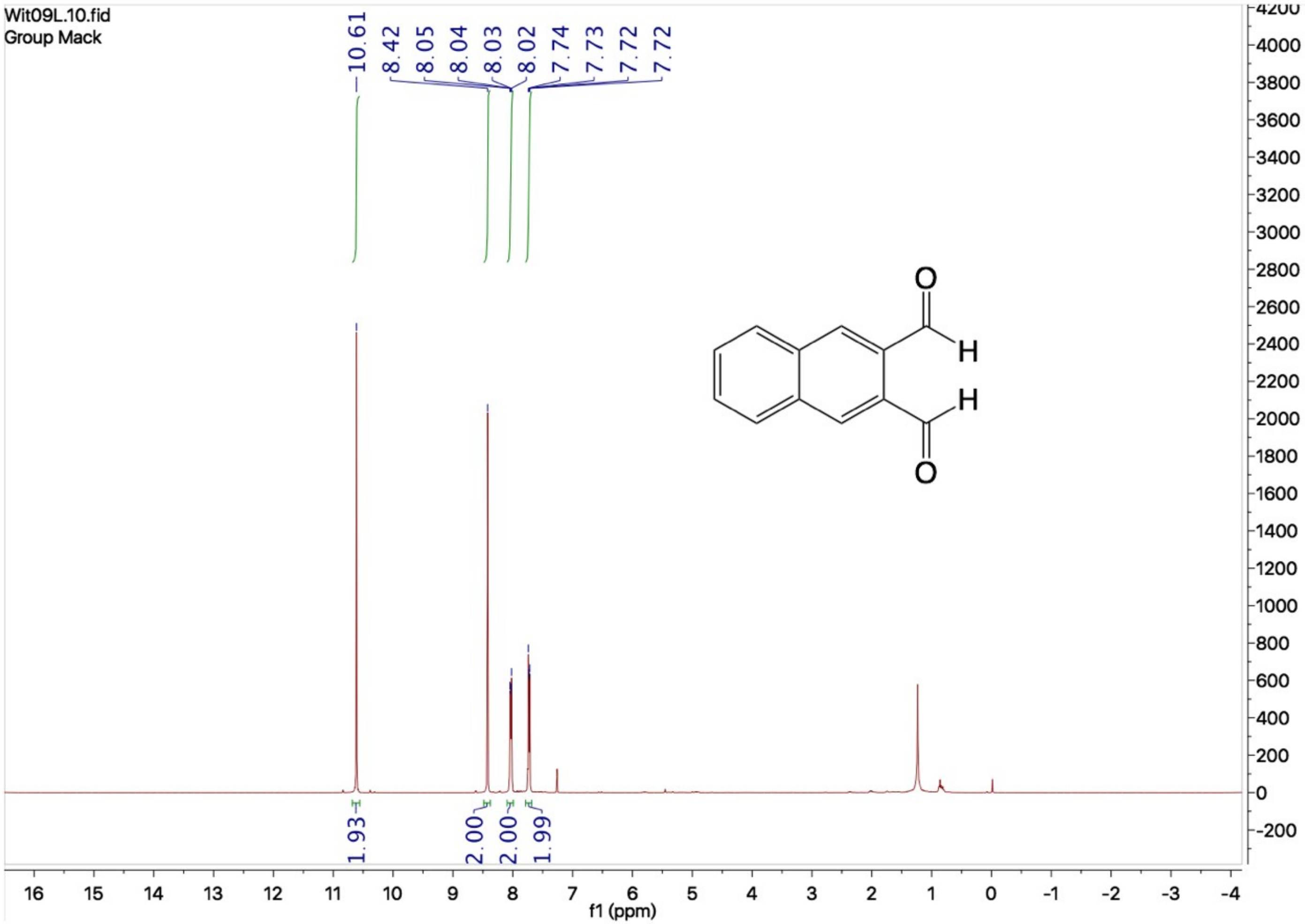

^{13}C-NMR Naphthalene-2,3-dicarbaldehyde

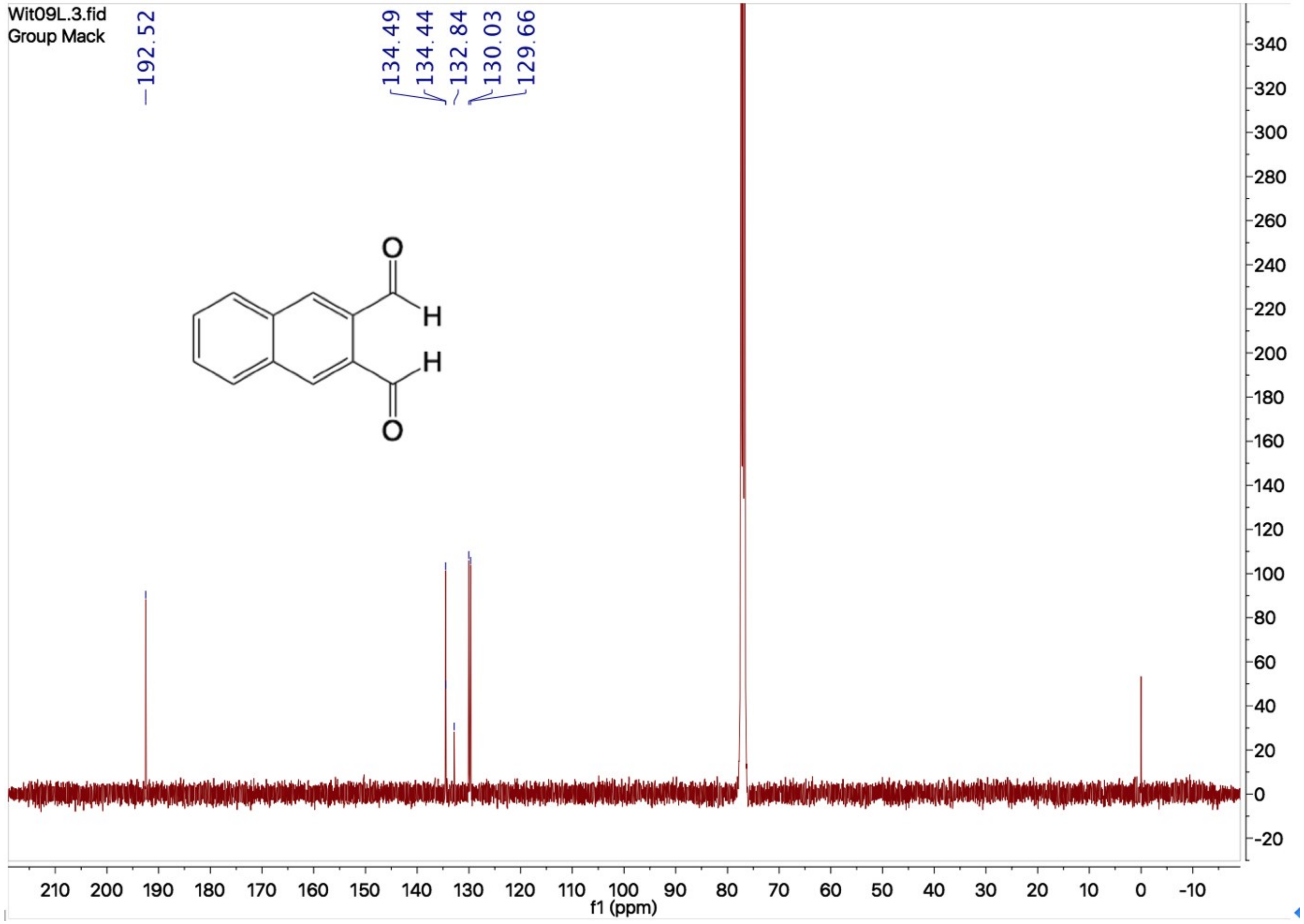

GCMS of Naphthalene-2,3-dicarbaldehyde

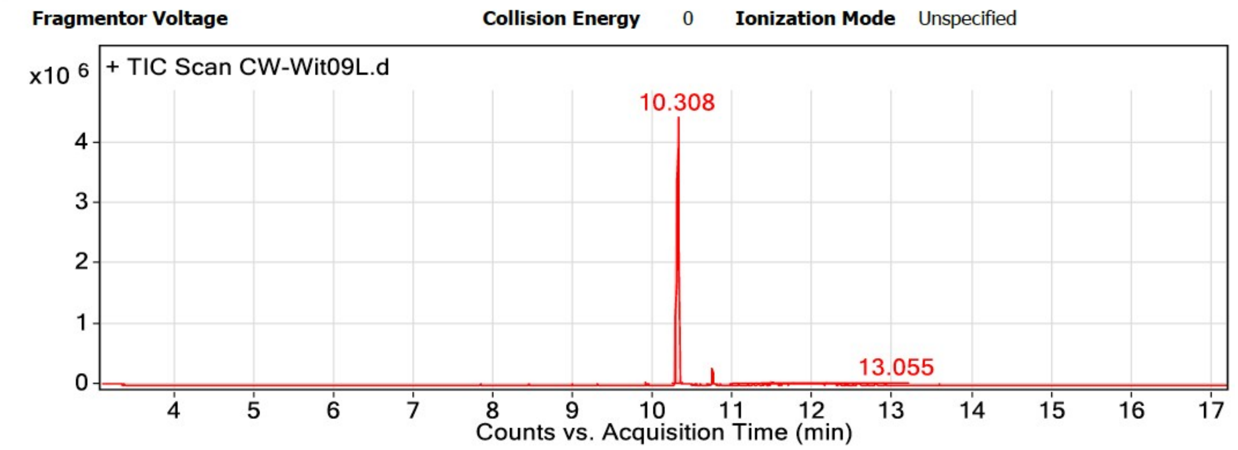

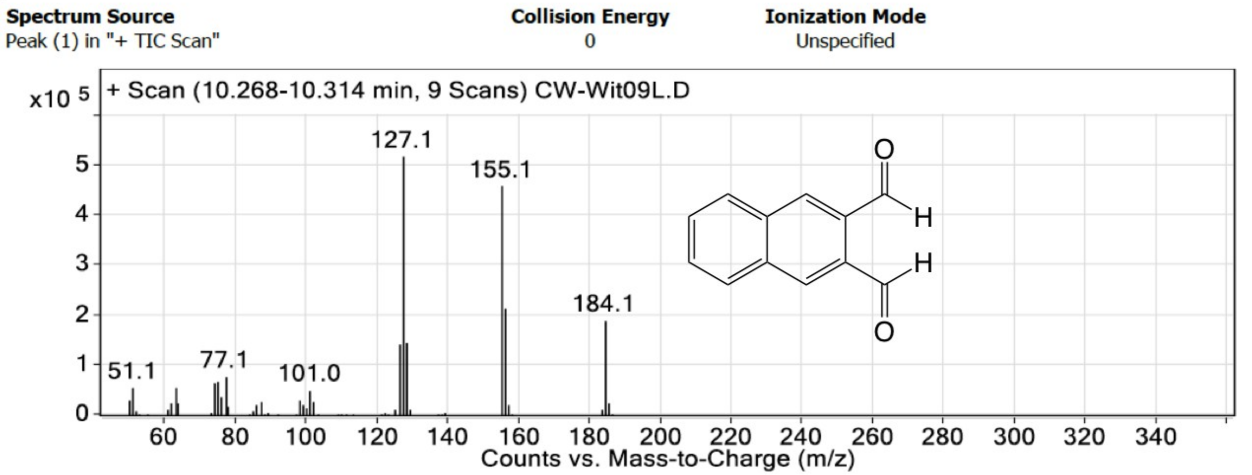

^{1}H-NMR of Anthracene-2,3-dicarboxylic acid diethyl ester

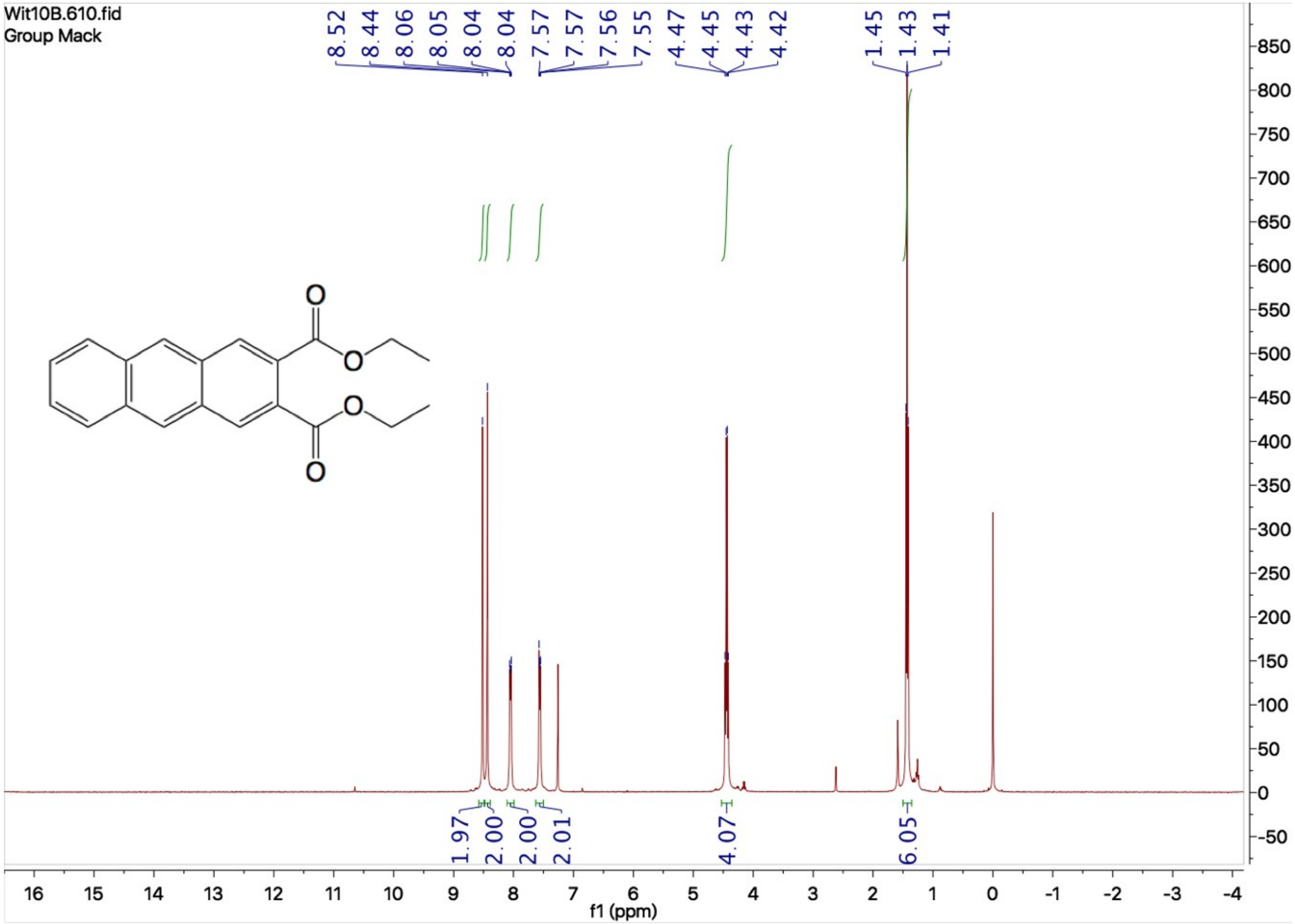

^{13}C-NMR of Anthracene-2,3-dicarboxylic acid diethyl ester

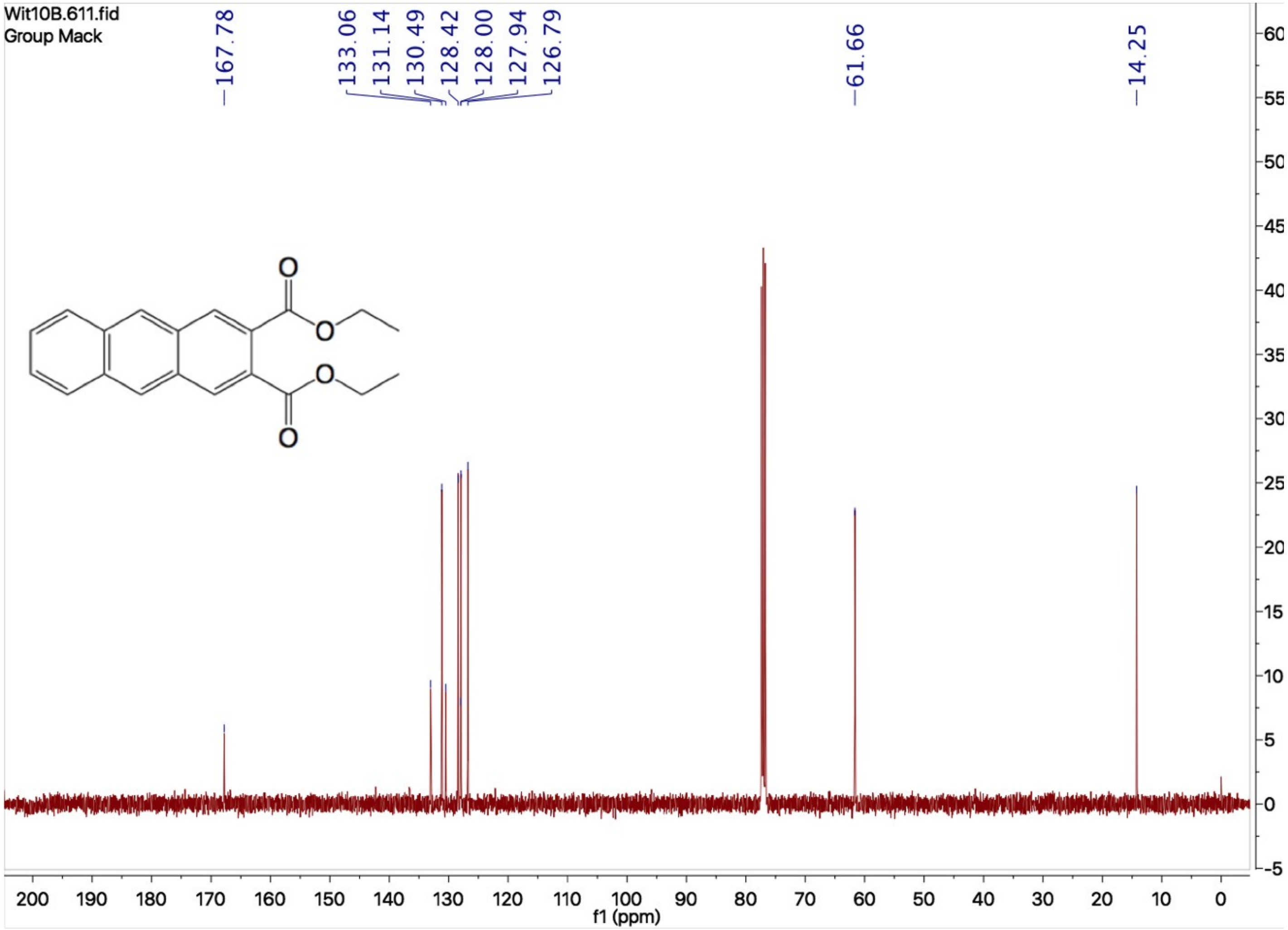

GCMS of Anthracene-2,3-dicarboxylic acid diethyl ester

Fragmentor Voltage **Collision Energy** 0 **Ionization Mode** Unspecified

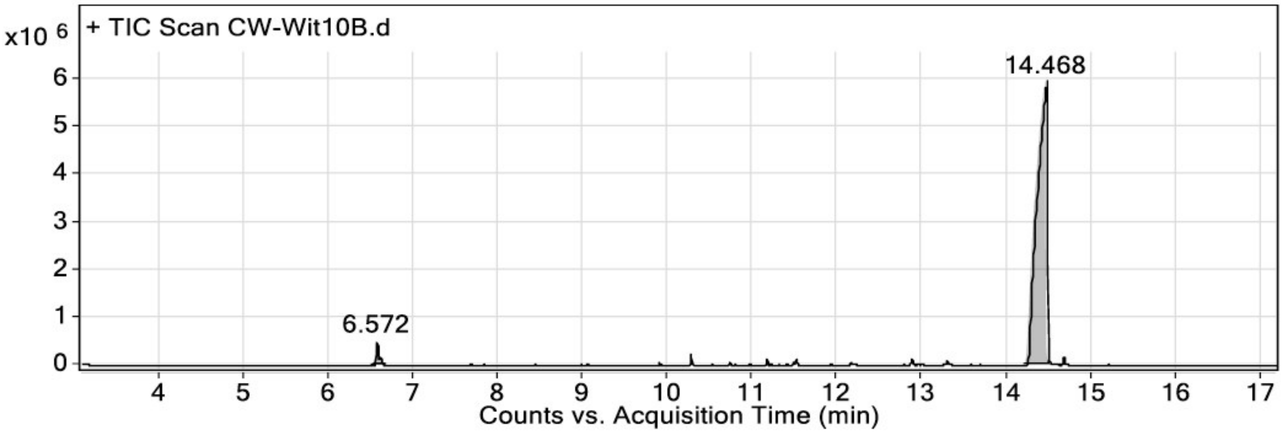

Spectrum Source Peak (2) in "+ TIC Scan" **Collision Energy** 0 **Ionization Mode** Unspecified

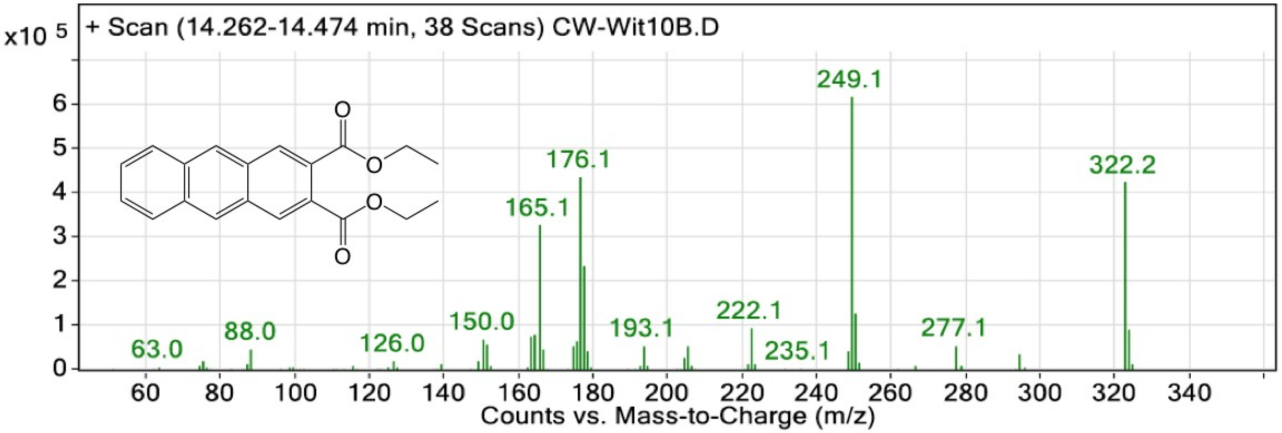

^{1}H-NMR of (3-Hydroxymethyl-anthracen-2-yl)-methanol

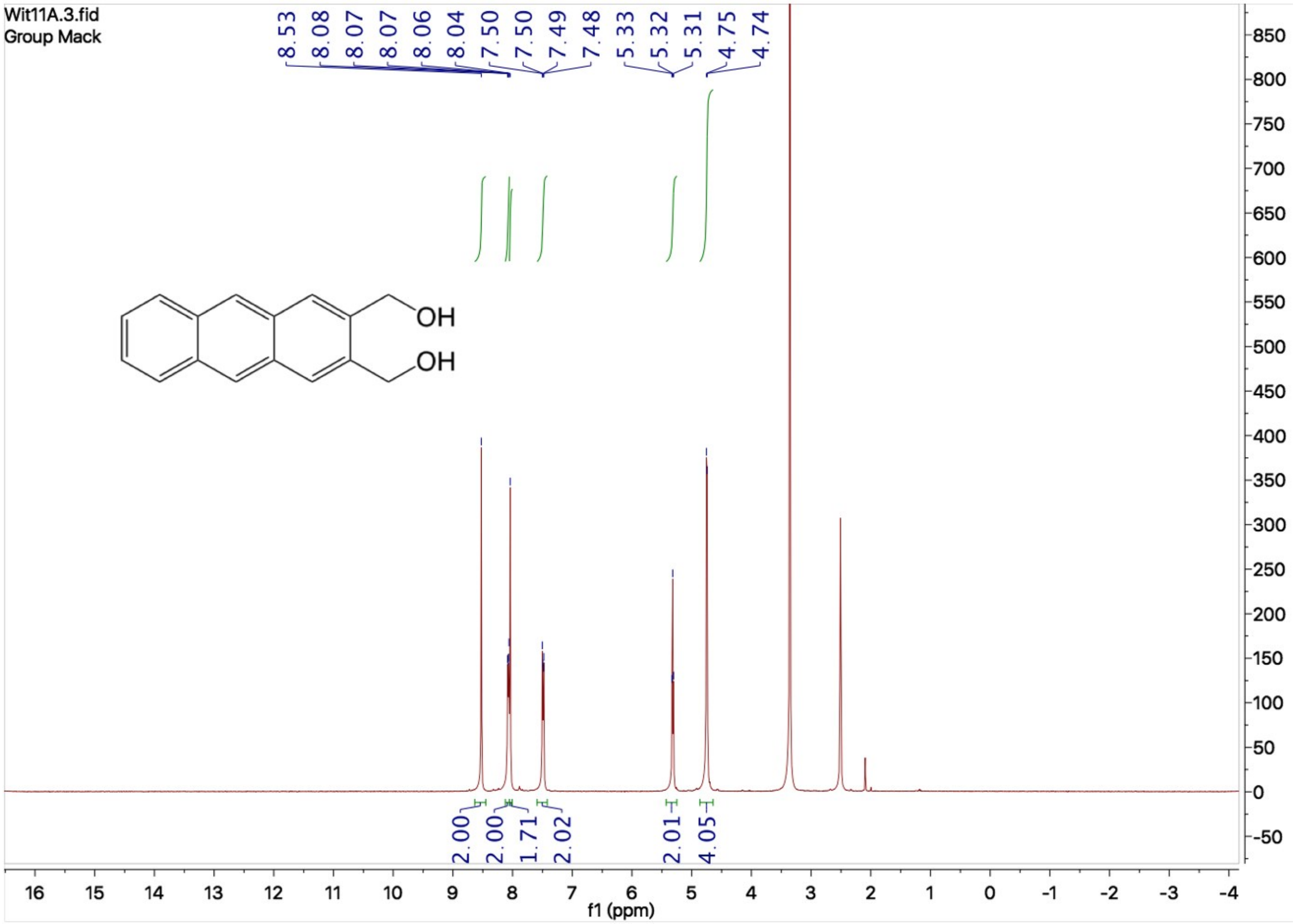

^{13}C-NMR of (3-Hydroxymethyl-anthracen-2-yl)-methanol

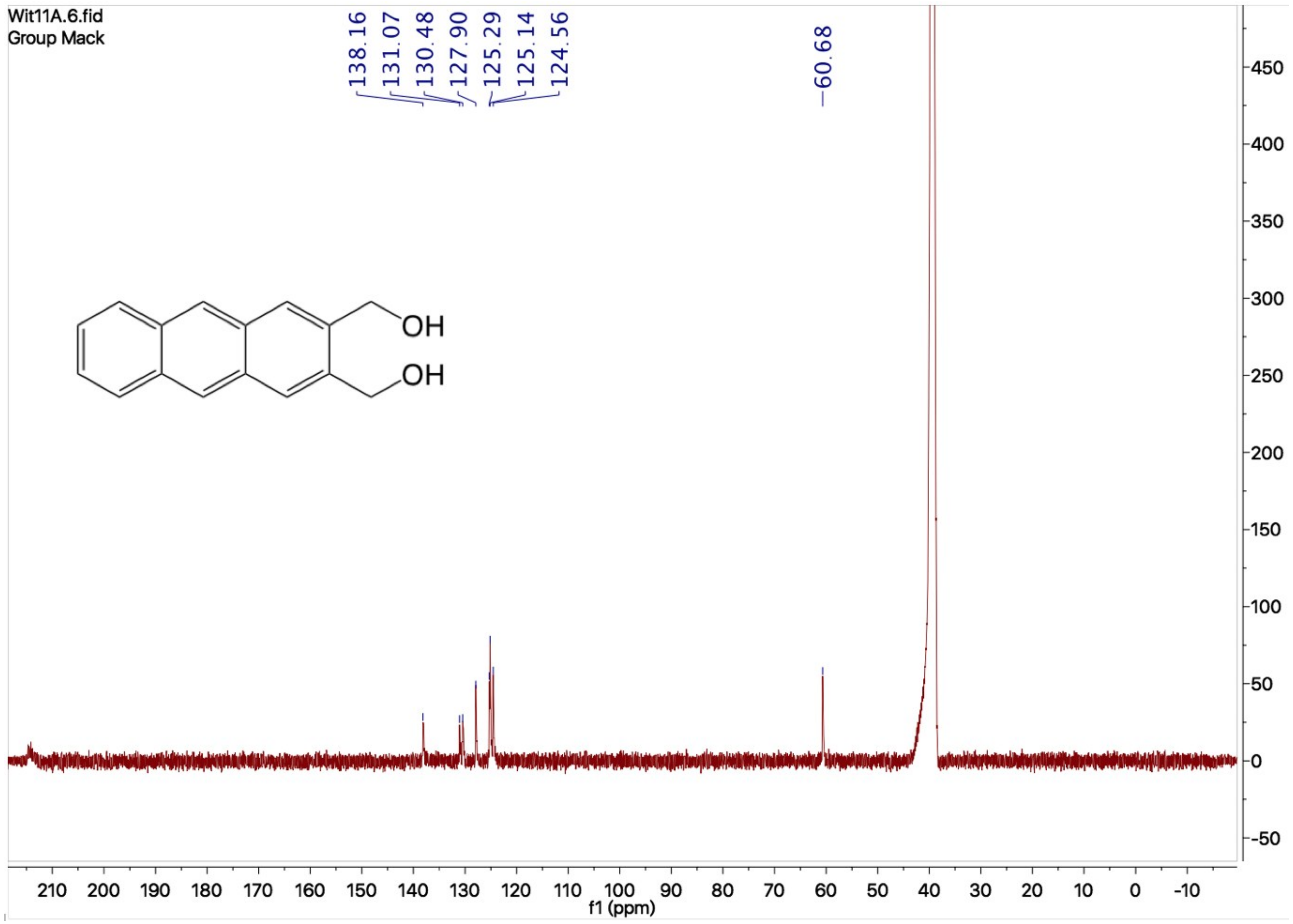

HRMS of (3-Hydroxymethyl-anthracen-2-yl)-methanol, (M+Na$^+$), $(C_{16}H_{14}O_2)Na^+$

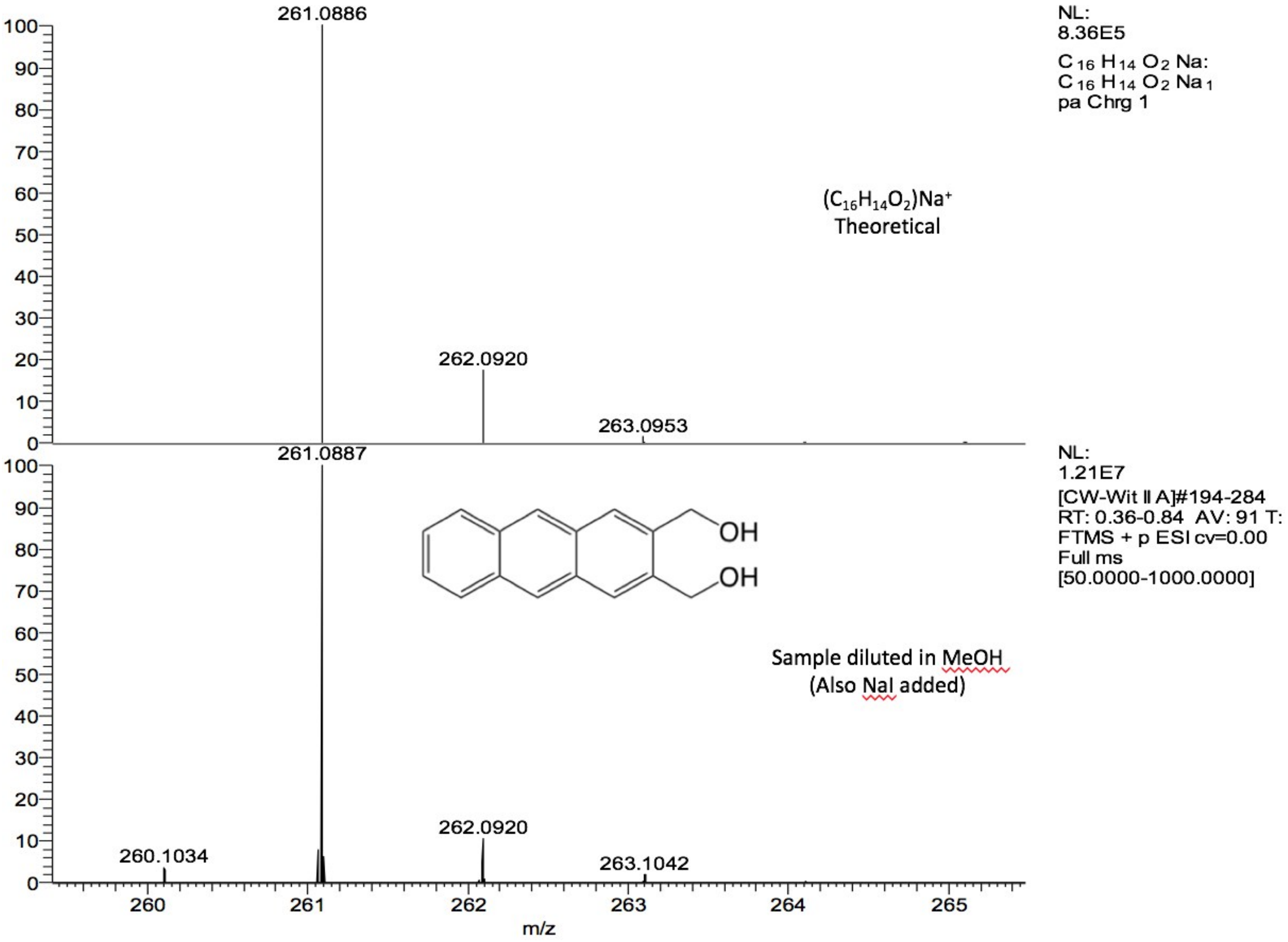

^{1}H-NMR of Anhracene-2,3-dicarbaldehyde

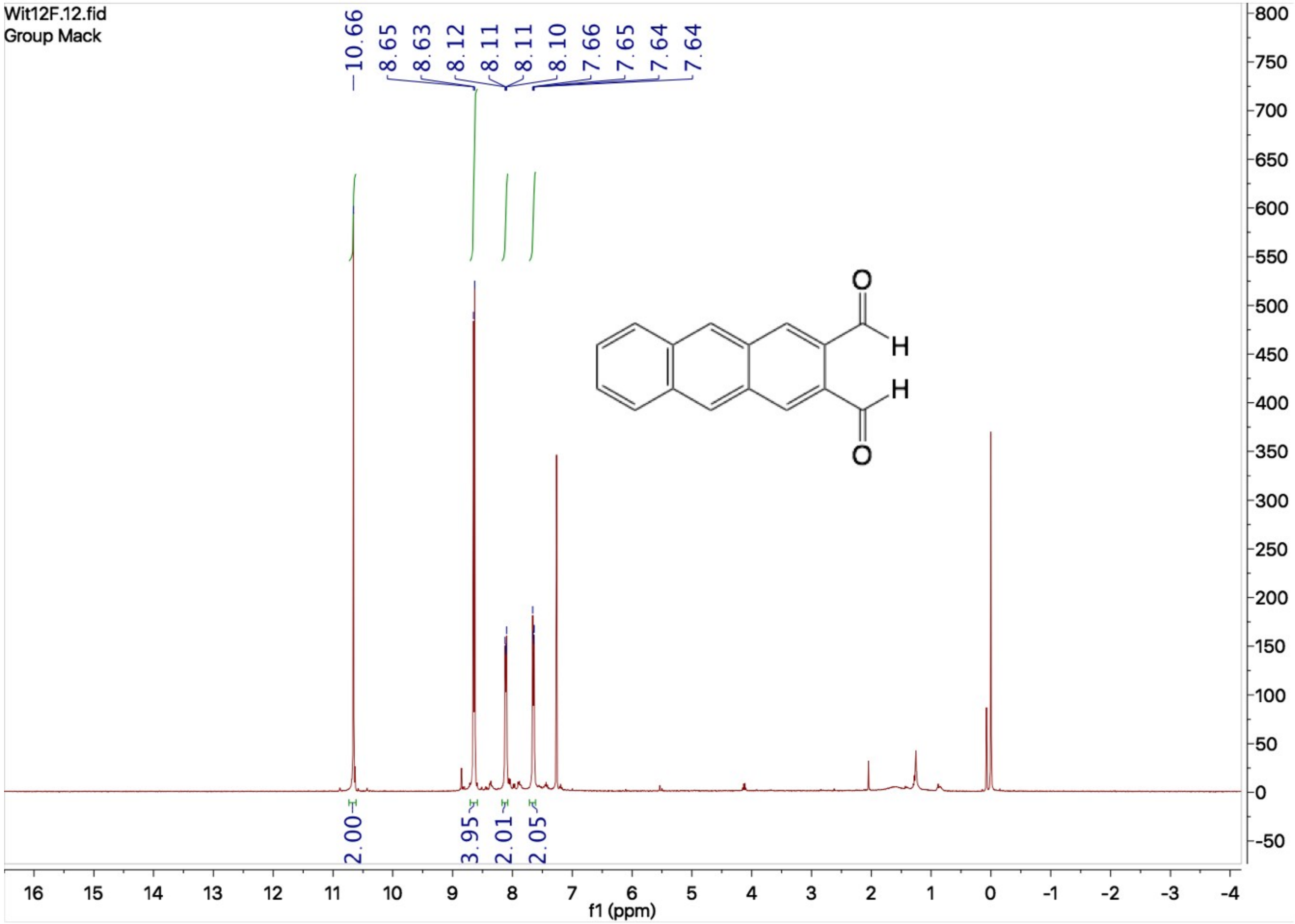

^{13}C-NMR of Anhracene-2,3-dicarbaldehyde

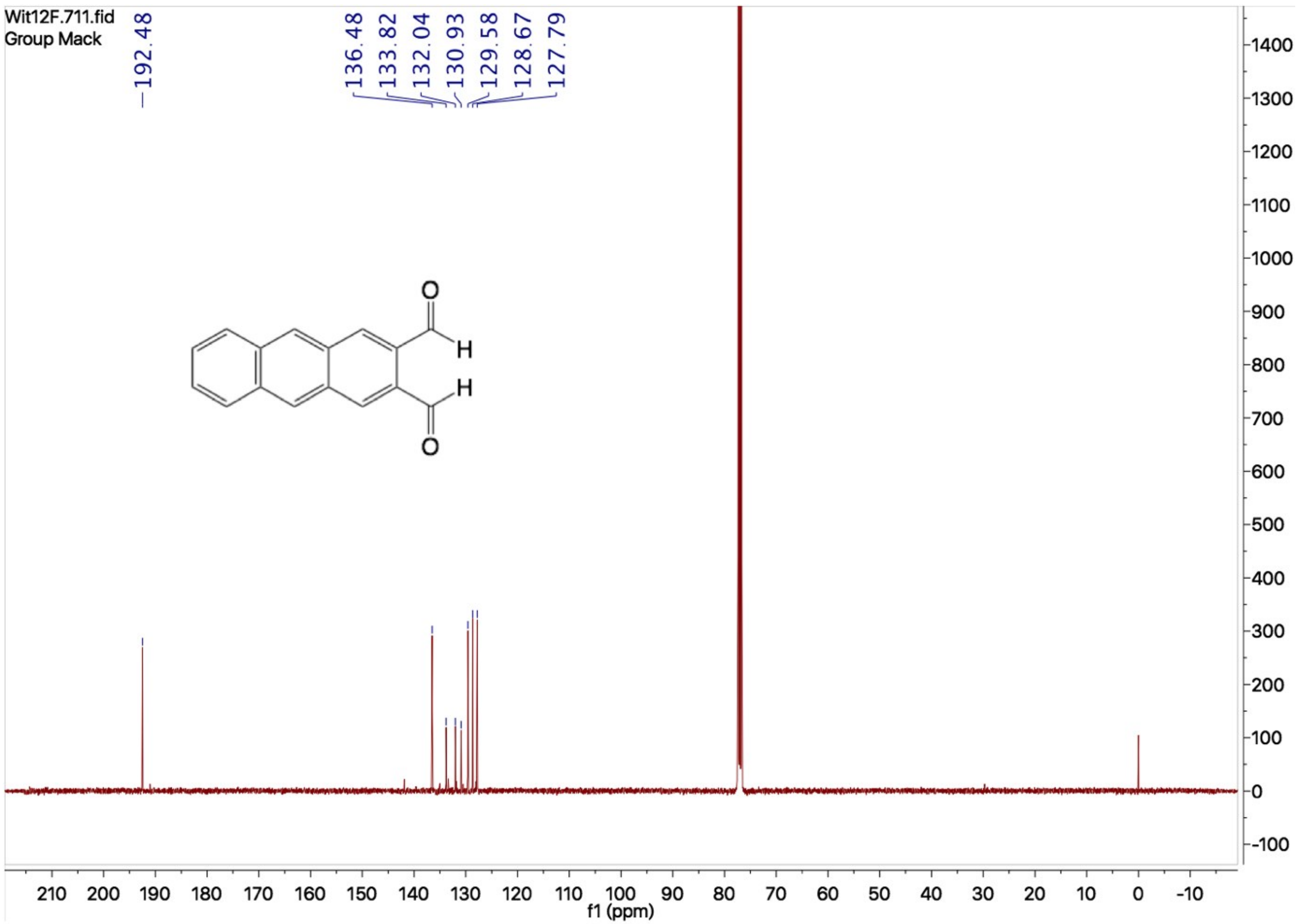

GCMS of Anhracene-2,3-dicarbaldehyde

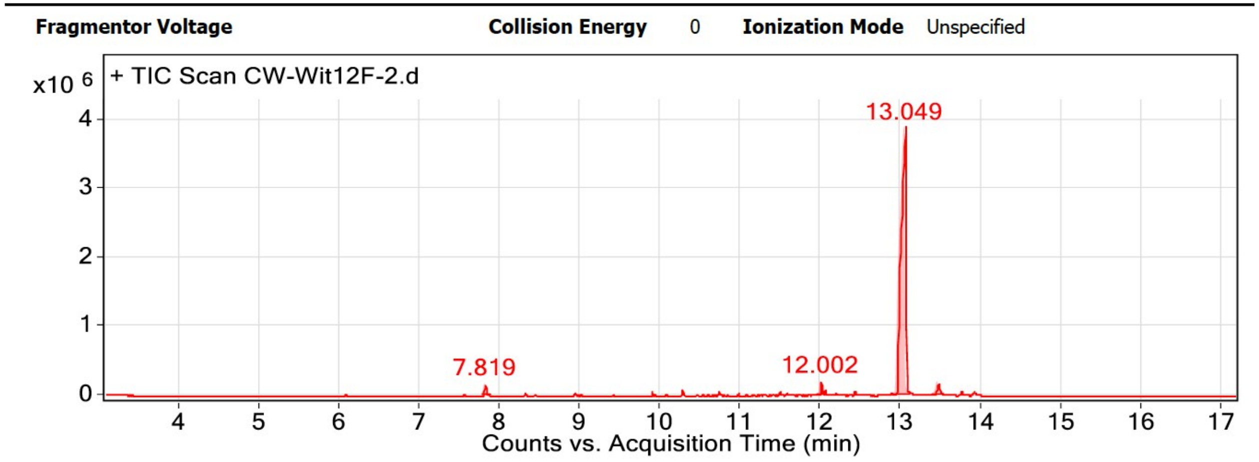

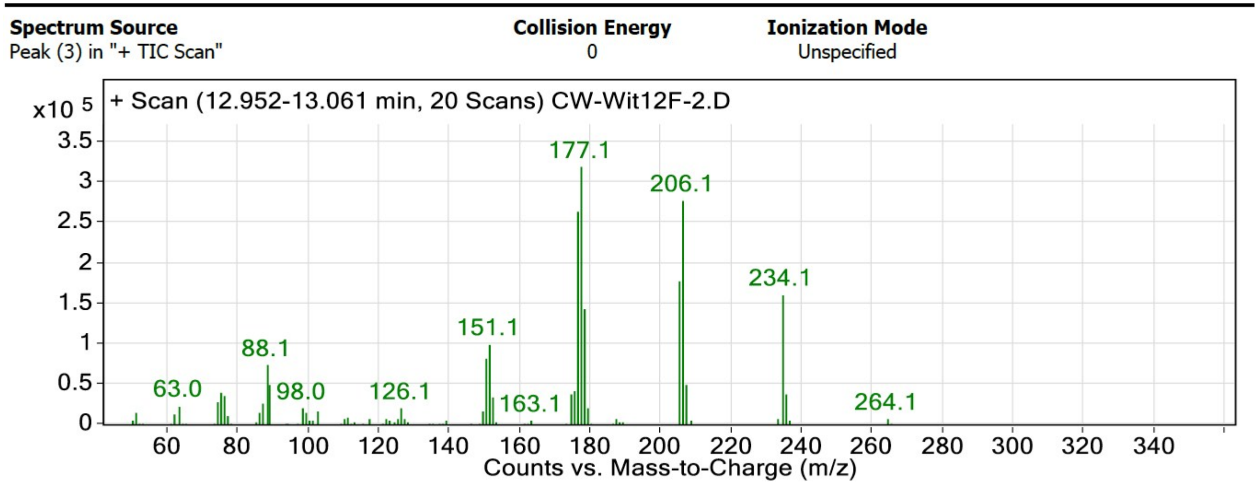

^{1}H-NMR of Naphthacene-2,3-dicarboxylic acid diethyl ester

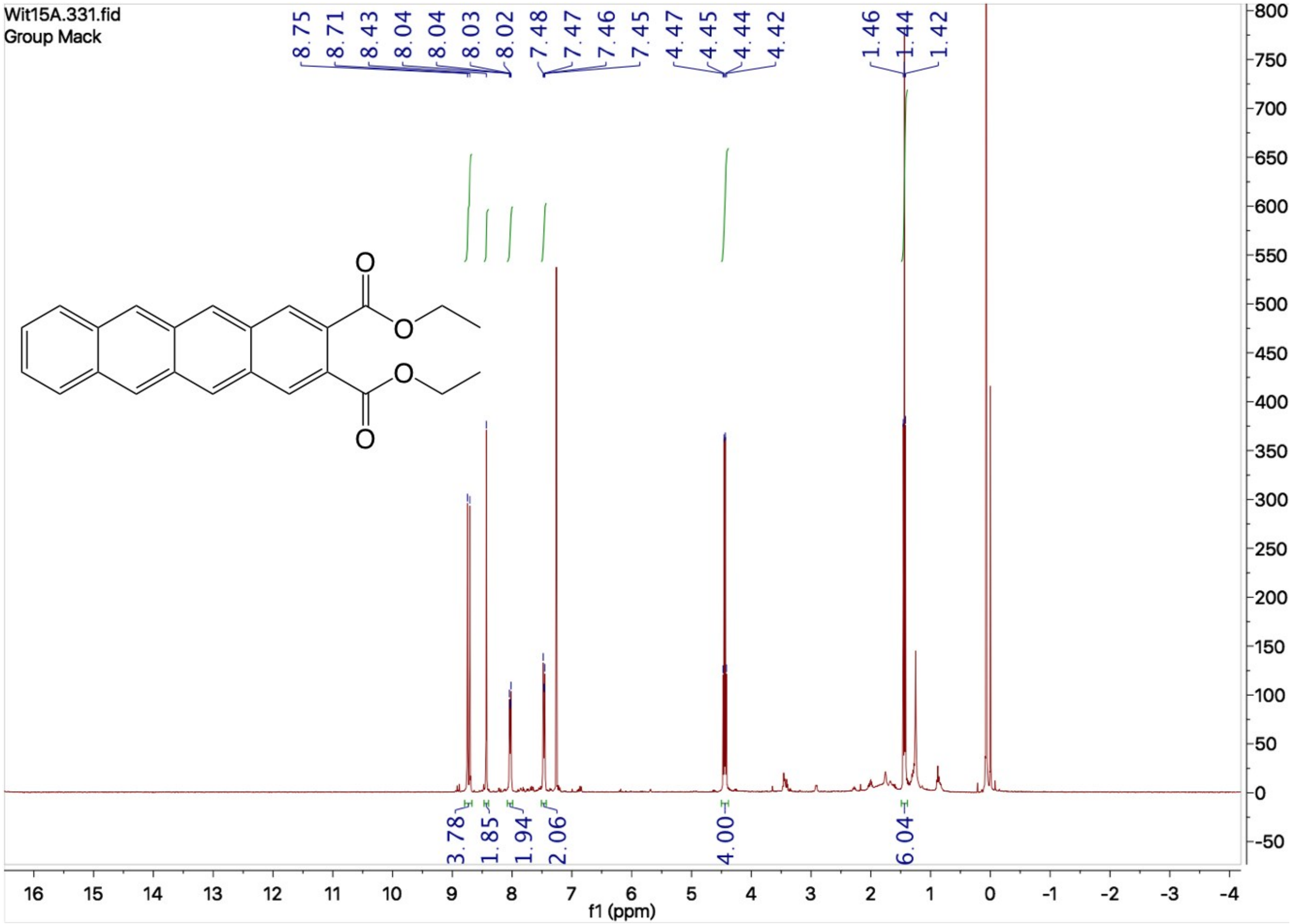

^{13}C-NMR of Naphthacene-2,3-dicarboxylic acid diethyl ester

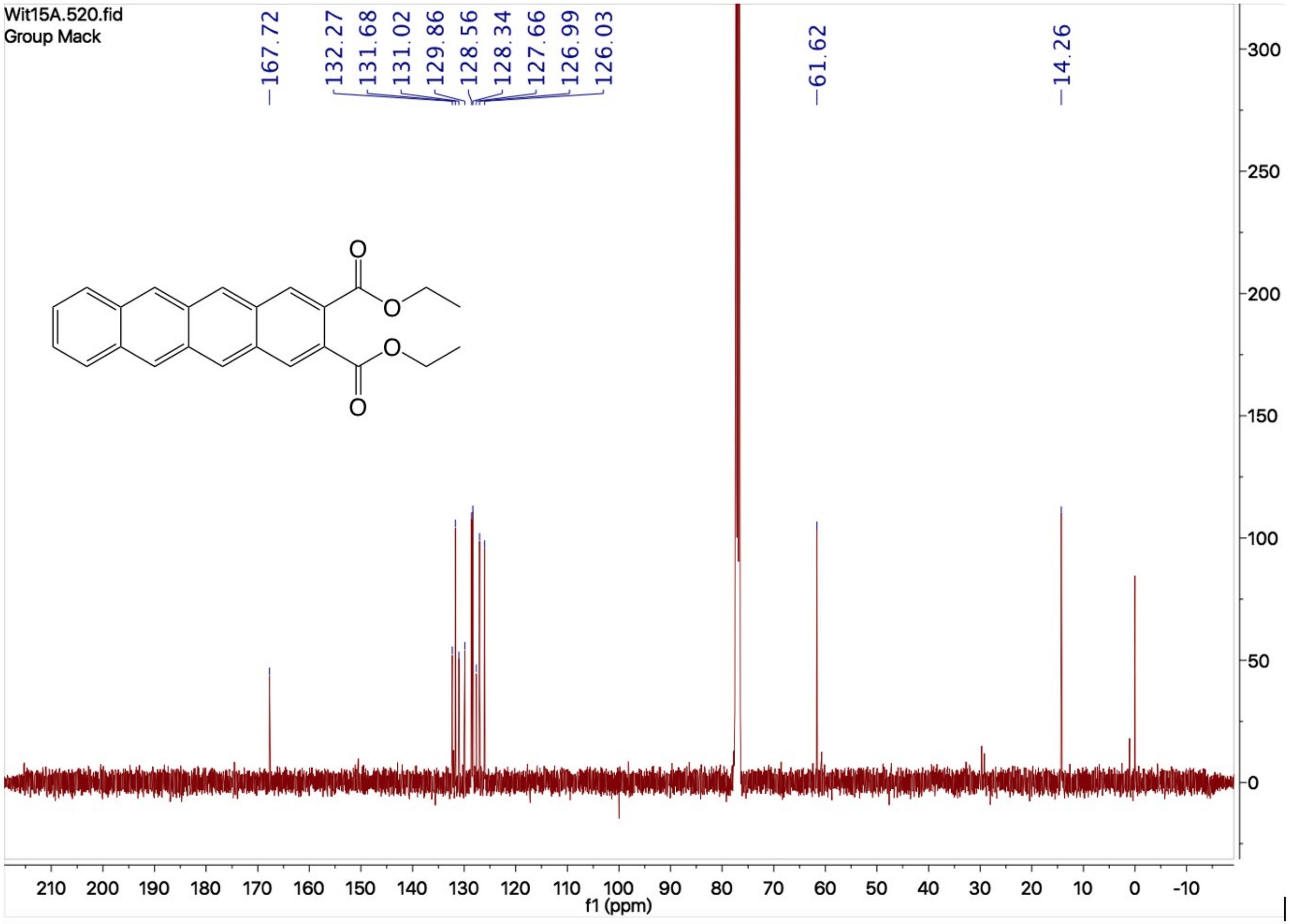

HRMS of Naphthacene-2,3-dicarboxylic acid diethyl ester, ($M+Na^+$), ($(C_{24}H_{20}O_4)Na^+$)

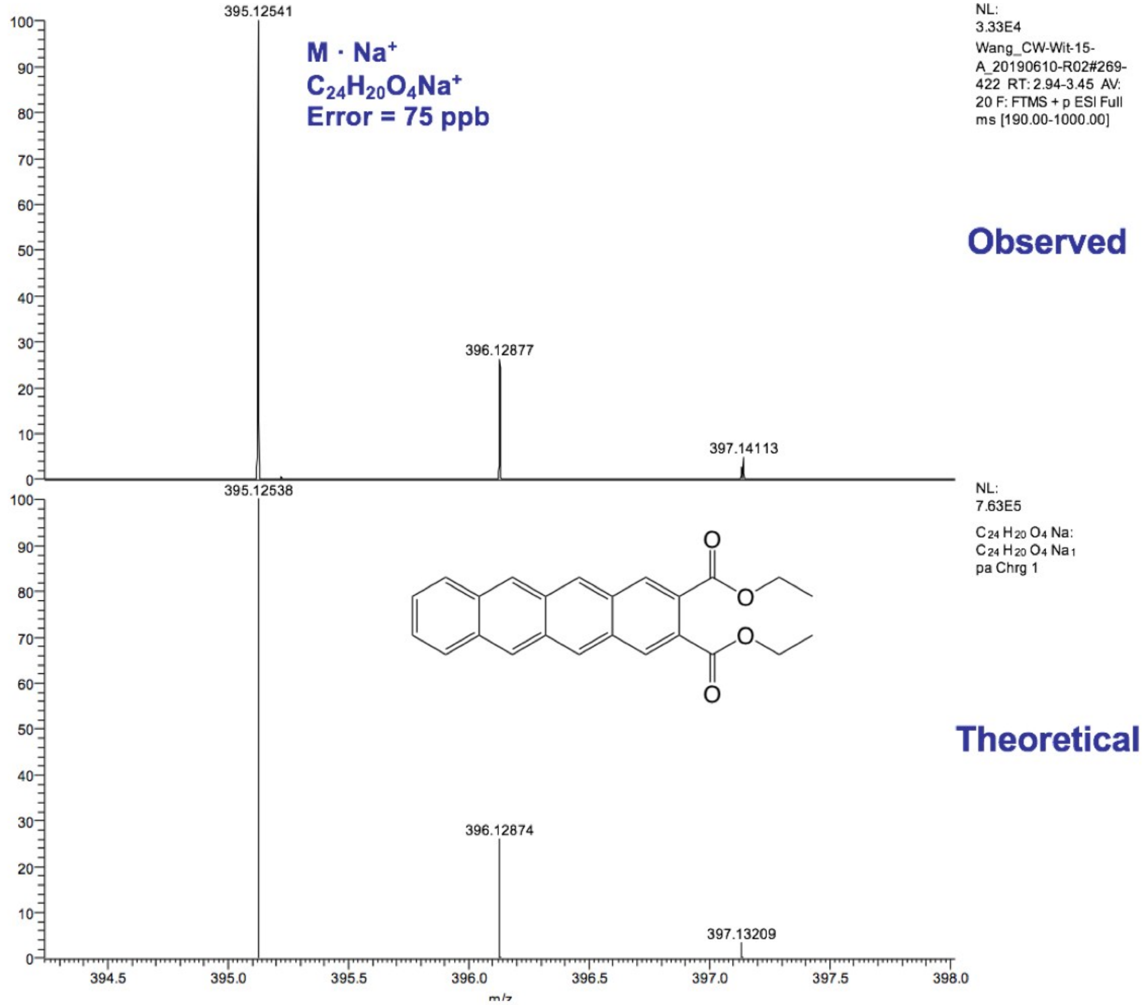

Eco-Scale Calculation

Eco-Scale is a standard to evaluate the safety and environmental impact.[8] We did this calculation to compare our method with previous research.[1]

Summery, (detailed calculation in the following)

Product	Eco-scale score		Price for every 10 mmol product	
	Previous research published by Lin et. al.[1]	Our process	Previous research published by Lin et. al.[1]	Our process
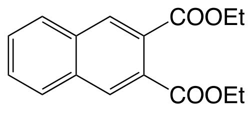	43	64	$52.7	$19.3
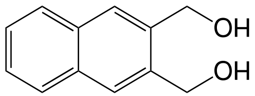	58	61.5	$129.3	$30.1
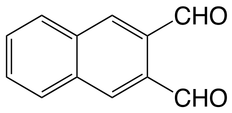	52.5	77.5	$210.3	$45.2
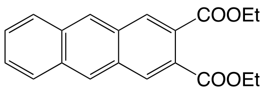	51	61	$281.4	$77.1

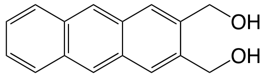	58.5	67.5	$441.6	$146.4
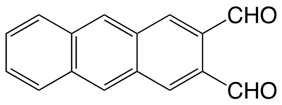	51.5	82.5	$601.1	$188.4
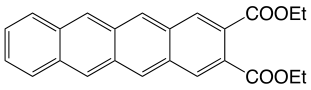	35.5	37	$1150.48	$955.7

From this form, we can see that, for every step, our process is more environment friendly based on Eco-scale point. And for every step to get same amount of product, our price is cheaper.

For the total seven steps to get Naphthacene-2,3-dicarboxylic acid diethyl ester

	Previous research[1]		For our process	
Parameter	Penalty points	Percentage in total penalty points(%)	Penalty points	Percentage in total penalty points(%)
Yield	73	20.9	121	48
Price of reaction components (to obtain 10 mmol of end product)	70	20	29	11.5
Safety	110	31.4	40	15.9
Technical set-up	14	4	0	0
Temperature/time	32	9.1	3	1.2
Workup and purification	51	14.6	59	23.4
Total penalty points	350		252	

For the previous research published by Lin et. al.[1], wittig reaction generating Naphthalene-2,3-dicarboxylic acid diethyl ester

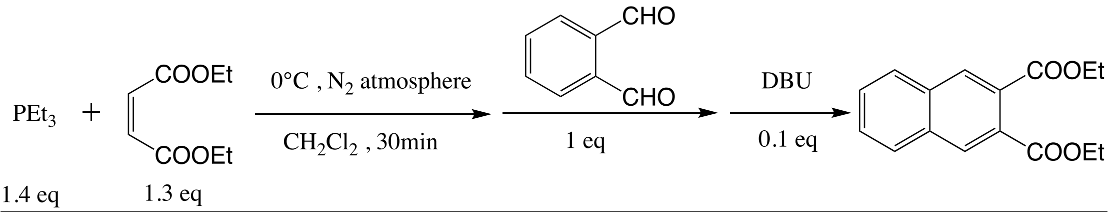

Parameter	Item	Penalty points	Note
Yield	86%	7	
Price of reaction components (to obtain 10 mmol of end product)	1,2-Phthalic dicarboxaldehyde	3	
	Triethylphosphine	3	
	Diethyl maleate	0	
	1,8-Diazabicyclo[5.4.0]undec-7-ene 2,3,4,6,7,8,9,10-Octahydropyrimido[1,2-a]azepine (DBU)	0	
	Dichloromethane	0	
	Water	0	

Safety	1,2-Phthalic dicarboxaldehyde	10	dangerous for evironment / toxic
	Triethylphosphine	5	highly flammable
	Diethyl maleate	0	
	1,8-Diazabicyclo[5.4.0]undec-7-ene 2,3,4,6,7,8,9,10-Octahydropyrimido[1,2-a]azepine (DBU)	10	dangerous for evironment / toxic
	Dichloromethane	0	
	Water	0	
Technical set-up	Instruments for controlled addition of chemicals	1	syringe used, drop wisely
	(Inert) gas atmosphere	1	
Temperature/time	Cooling to 0°C	4	
Workup and purification	Liquid-liquid extraction	3	
	Classical chromatography	10	
Eco-scale point	100-57=43		

For the previous research published by Lin et. al.[1], reduction generating (3-Hydroxymethyl-naphthalen-2-yl)-methanol

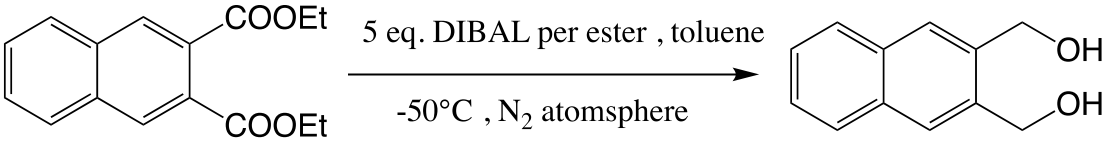

Parameter	Item	Penalty points	Note
Yield	86%	7	
Price of reaction components (to obtain 10 mmol of end product)	Naphthalene-2,3-dicarboxylic acid diethyl ester	5	Lab-made starting material, \$61.3 Based on previous calculation
	DIBAL	5	
Safety	Naphthalene-2,3-dicarboxylic acid diethyl ester	5	Lab-made starting material, dangerous for evironment.
	DIBAL	10	Highly flammable, Toxic

Technical set-up	Instruments for controlled addition of chemicals	1	syringe used, drop wisely
	(Inert) gas atmosphere	1	
Temperature/time	Cooling, < 0°C	5	-50°C used
Workup and purification	Liquid-liquid extraction	3	
Eco-scale point	100-42=58		

For the previous research published by Lin et. al.[1], oxidation generating Naphthalene-2,3-dicarbaldehyde

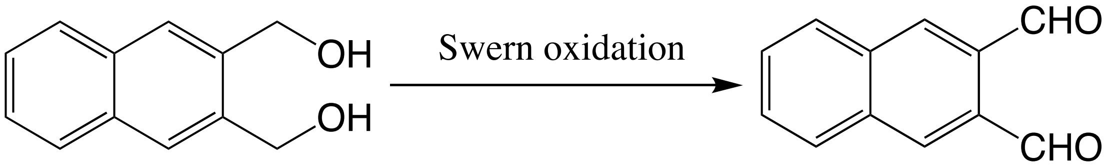

Parameter	Item	Penalty points	Note
Yield	83%	8.5	
Price of reaction components (to obtain 10 mmol of end product)	2,3-Bis(hydroxymethyl)naphthalene	5	Lab-made starting material, $114.8 Based on previous calculation
	Oxalyl chloride	3	
	DMSO	3	
	Triethylamine	3	
Safety	2,3-Bis(hydroxymethyl)naphthalene	N/A	Lab-made starting material, safety information not found
	Oxalyl chloride	5	toxic
	DMSO	0	
	Triethylamine	10	Highly flammable, toxic
Technical set-up	Instruments for controlled addition of chemicals	1	syringe used, drop wisely
	(Inert) gas atmosphere	1	
Temperature/time	Cooling, < 0°C	5	-80°C used
Workup and purification	Liquid-liquid extraction	3	
Eco-scale point	100-44.5=52.5		

For the previous research published by Lin et. al.[1], wittig reaction generating Anthracene-2,3-dicarboxylic acid diethyl ester

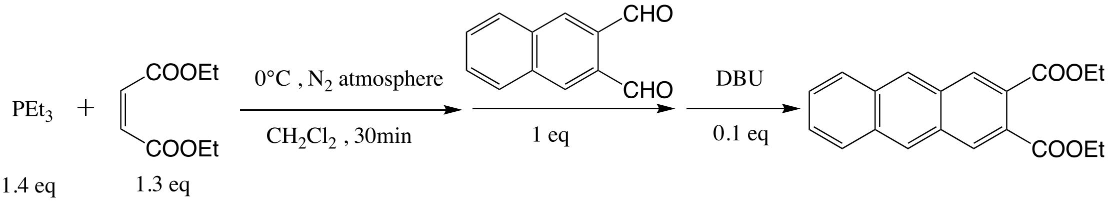

Parameter	Item	Penalty points	Note
Yield	86%	7	
Price of reaction components (to obtain 10 mmol of end product)	2,3-Naphthalenedicarboxaldehyde	5	Lab-made starting material, $244.5 Based on previous calculation
	Triethylphosphine	3	
	Diethyl maleate	0	
	1,8-Diazabicyclo[5.4.0]undec-7-ene 2,3,4,6,7,8,9,10-Octahydropyrimido[1,2-a]azepine (DBU)	0	
	Dichloromethane	0	
	Water	0	
Safety	2,3-Naphthalenedicarboxaldehyde	0	
	Triethylphosphine	5	highly flammable
	Diethyl maleate	0	
	1,8-Diazabicyclo[5.4.0]undec-7-ene 2,3,4,6,7,8,9,10-Octahydropyrimido[1,2-a]azepine (DBU)	10	dangerous for evironment / toxic
	Dichloromethane	0	
	Water	0	
Technical set-up	Instruments for controlled addition of chemicals	1	syringe used, dropwisely
	(Inert) gas atmosphere	1	
Temperature/time	Cooling to 0°C	4	
Workup and	Liquid-liquid extraction	3	

purification			
	Classical chromatography	10	
Eco-scale point	100-49=51		

For the previous research published by Lin et. al.[1], reduction generating (3-Hydroxymethyl-anthracen-2-yl)-methanol

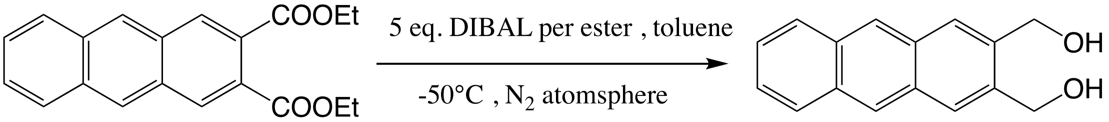

Parameter	Item	Penalty points	Note
Yield	77%	11.5	
Price of reaction components (to obtain 10 mmol of end product)	Anthracene-2,3-dicarboxylic acid diethyl ester	5	Lab-made starting material, $303.6 Based on previous calculation
	DIBAL	5	
Safety	Anthracene-2,3-dicarboxylic acid diethyl ester	N/A	Lab-made starting material, safety information not found
	DIBAL	10	Highly flammable, Toxic
Technical set-up	Instruments for controlled addition of chemicals	1	syringe used, dropwisely
	(Inert) gas atmosphere	1	
Temperature/time	Cooling, < 0°C	5	-50°C used
Workup and purification	Liquid-liquid extraction	3	
Eco-scale point	100-41.5=58.5		

For the previous research published by Lin et. al.[1], oxidation generating Anhracene-2,3-dicarbaldehyde

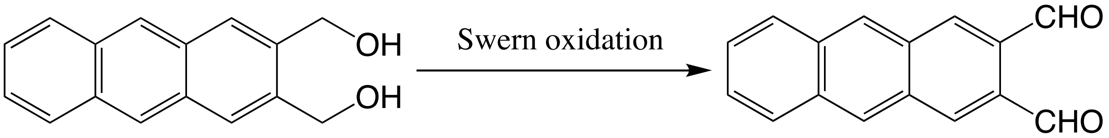

Parameter	Item	Penalty points	Note
Yield	81%	9.5	

Price of reaction components (to obtain 10 mmol of end product)	2,3-Bis(hydroxymethyl)naphthalene	5	Lab-made starting material, $545.2 Based on previous calculation
	Oxalyl chloride	3	
	DMSO	3	
	Triethylamine	3	
Safety	2,3-Bis(hydroxymethyl)naphthalene	N/A	Lab-made starting material, safety information not found
	Oxalyl chloride	5	toxic
	DMSO	0	
	Triethylamine	10	Highly flammable, toxic
Technical set-up	Instruments for controlled addition of chemicals	1	syringe used, drop wisely
	(Inert) gas atmosphere	1	
Temperature/time	Cooling, < 0°C	5	-80°C used
Workup and purification	Liquid-liquid extraction	3	
Eco-scale point	100-48.5=51.5		

For the previous research published by Lin et. al.[1], wittig reaction generating Naphthacene-2,3-dicarboxylic acid diethyl ester

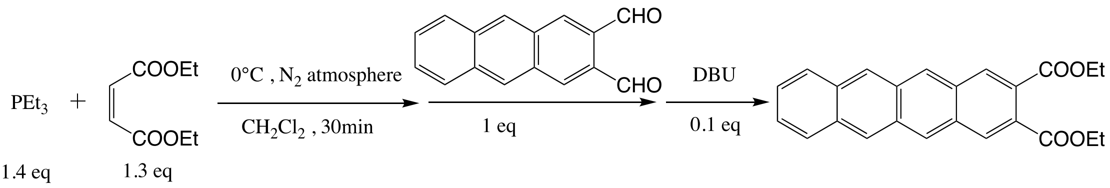

Parameter	Item	Penalty points	Note
Yield	55%	22.5	
Price of reaction components (to obtain 10 mmol of end product)	Anthracene-2,3-dicarbaldehyde	5	Lab-made starting material, $1092.9 Based on previous calculation
	Triethylphosphine	3	
	Diethyl maleate	0	
	1,8-Diazabicyclo[5.4.0]undec-7-ene 2,3,4,6,7,8,9,10-Octahydropyrimido[1,2-	0	

	a]azepine (DBU)		
	Dichloromethane	0	
	Water	0	
Safety	Anthracene-2,3-dicarbaldehyde	N/A	Lab-made starting material, safety information not found
	Triethylphosphine	5	highly flammable
	Diethyl maleate	0	
	1,8-Diazabicyclo[5.4.0]undec-7-ene 2,3,4,6,7,8,9,10-Octahydropyrimido[1,2-a]azepine (DBU)	10	dangerous for evironment / toxic
	Dichloromethane	0	
	Water	0	
Technical set-up	Instruments for controlled addition of chemicals	1	syringe used, dropwisely
	(Inert) gas atmosphere	1	
Temperature/time	Cooling to 0°C	4	
Workup and purification	Liquid-liquid extraction	3	
	Classical chromatography	10	
Eco-scale point	100-64.5=35.5		

For our process, wittig reaction generating Naphthalene-2,3-dicarboxylic acid diethyl ester

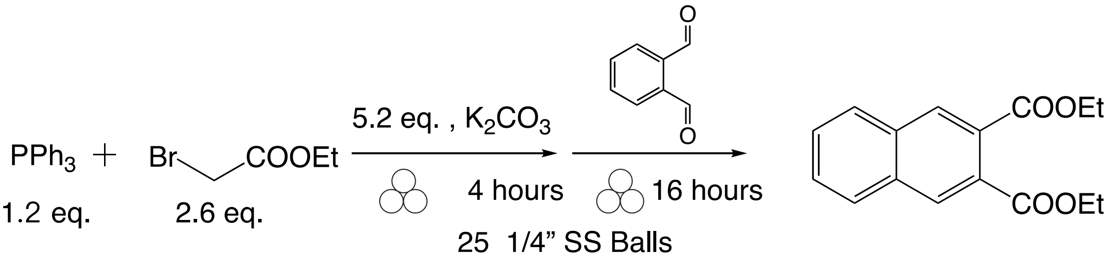

Parameter	Item	Penalty points	Note
Yield	84%	8	
Price of reaction components (to obtain 10 mmol of end product)	1,2-Phthalic dicarboxaldehyde	3	
	Triphnyl phosphine	0	
	Ethyl bromoacetate	0	
	Potassium carbonate	0	
Safety	1,2-Phthalic dicarboxaldehyde	10	dangerous for evironment / toxic
	Triphnyl phosphine	0	
	Ethyl bromoacetate	5	toxic
	Potassium carbonate	0	
Technical set-up	Common setup	0	
Temperature/time	Room temperature, < 24 h	0	
Workup and purification	Simple filtration	0	
	Classical chromatography	10	
Eco-scale point	100-36=64		

For our process, Reduction generating (3-Hydroxymethyl-naphthalen-2-yl)-methanol

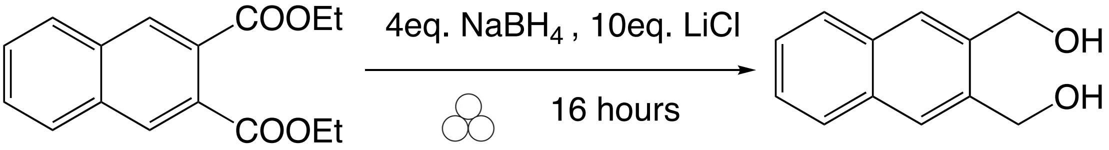

Parameter	Item	Penalty points	Note

Yield	75%	12.5	
Price of reaction components (to obtain 10 mmol of end product)	Naphthalene-2,3-dicarboxylic acid diethyl ester	3	Lab-made starting material, $25.7 Based on previous calculation
	Sodium borohydride	0	
	Lithium chloride	0	
Safety	Naphthalene-2,3-dicarboxylic acid diethyl ester	5	Lab-made starting material, dangerous for evironment.
	Sodium borohydride	5	Toxic
	Lithium chloride	0	
Technical set-up	Common setup	0	
Temperature/time	Room temperature, < 24 h	0	
Workup and purification	Liquid-liquid extraction	3	
	Classical chromatography	10	
Eco-scale point	100-38.5=61.5		

For our process, Oxidation generating Naphthalene-2,3-dicarbaldehyde

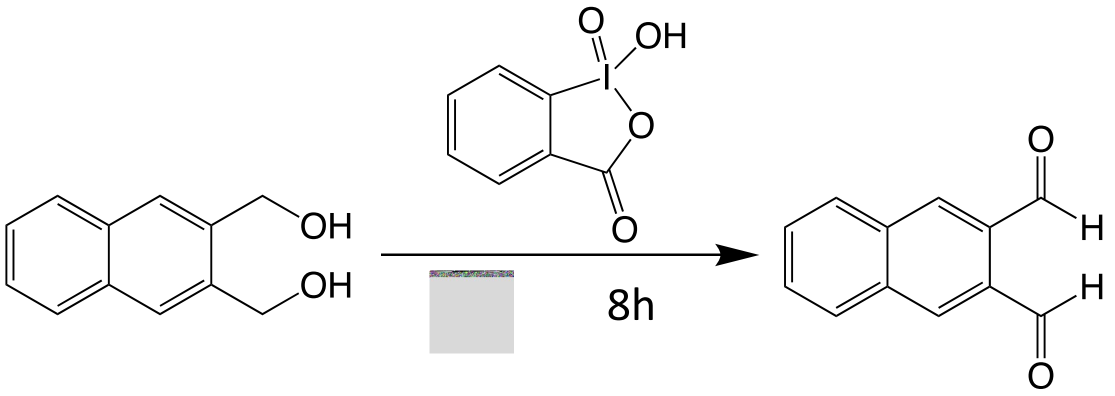

Parameter	Item	Penalty points	Note
Yield	81%	9.5	
Price of reaction components (to obtain 10 mmol of end product)	2,3-Bis(hydroxymethyl)naphthalene	3	Lab-made starting material, $37.5 Based on previous calculation

	IBX	0	Lab-made starting material, $7.7 based on preparation method
Safety	2,3-Bis(hydroxymethyl)naphthalene	N/A	Lab-made starting material, safety information not found
	IBX	0	
Technical set-up	Common setup	0	
Temperature/time	Room temperature, < 24 h	0	
Workup and purification	Classical chromatography	10	
Eco-scale point	100-22.5=77.5		

For our process, Wittig reaction generating Anthracene-2,3-dicarboxylic acid diethyl ester

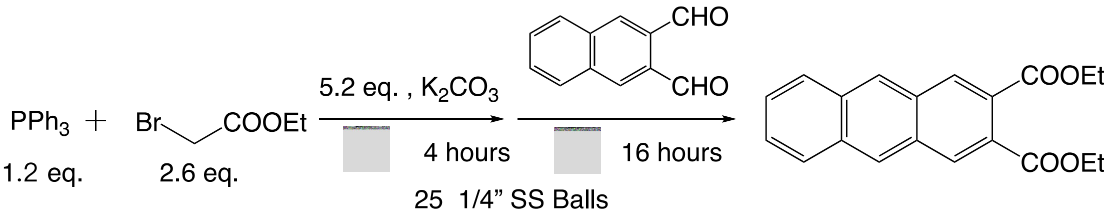

Parameter	Item	Penalty points	Note
Yield	62%	19	
Price of reaction components (to obtain 10 mmol of end product)	2,3-Naphthalenedicarboxaldehyde	5	Lab-made starting material, $72.9 Based on previous calculation
	Triphnyl phosphine	0	
	Ethyl bromoacetate	0	
	Potassium carbonate	0	
Safety	2,3-Naphthalenedicarboxaldehyde	0	
	Triphnyl phosphine	0	
	Ethyl bromoacetate	5	toxic
	Potassium carbonate	0	

Technical set-up	Common setup	0	
Temperature/time	Room temperature, < 24 h	0	
Workup and purification	Simple filtration	0	
	Classical chromatography	10	
Eco-scale point	100-39=61		

For our process, Reduction generating (3-Hydroxymethyl-anthracen-2-yl)-methanol

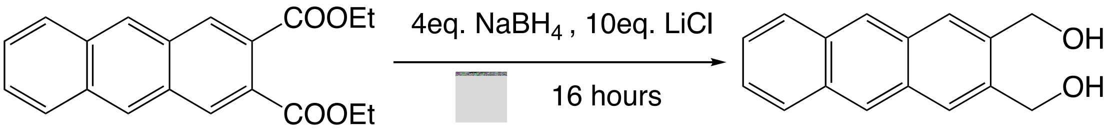

Parameter	Item	Penalty points	Note
Yield	55%	22.5	
Price of reaction components (to obtain 10 mmol of end product)	Anthracene-2,3-dicarboxylic acid diethyl ester	5	Lab-made starting material, \$140.2 Based on previous calculation
	Sodium borohydride	0	
	Lithium chloride	0	
Safety	Anthracene-2,3-dicarboxylic acid diethyl ester	N/A	Lab-made starting material, safety information not found
	Sodium borohydride	5	Toxic
	Lithium chloride	0	
Technical set-up	Common setup	0	
Temperature/time	Room temperature, < 24 h	0	
Workup and purification	Simple filtration	0	
Eco-scale point	100-41.5=67.5		

For our process, Oxidation generating Anhracene-2,3-dicarbaldehyde

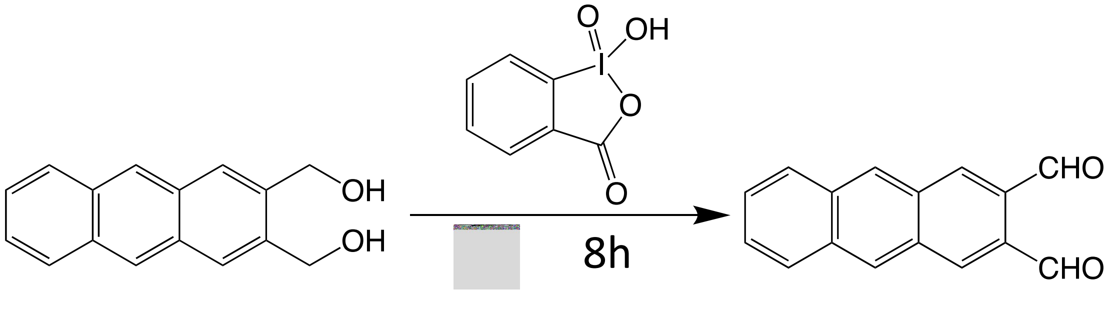

Parameter	Item	Penalty points	Note
Yield	81%	9.5	
Price of reaction components (to obtain 10 mmol of end product)	2,3-Bis(hydroxymethyl)naphthalene	5	Lab-made starting material, $180.7 Based on previous calculation
	IBX	0	Lab-made starting material, $7.7 based on preparation method
Safety	2,3-Bis(hydroxymethyl)naphthalene	N/A	Lab-made starting material, safety information not found
	IBX	0	
Technical set-up	Common setup	0	
Temperature/time	Room temperature, < 24 h	0	
Workup and purification	Simple filtration	0	
	Sublimation	3	
Eco-scale point	100-17.5=82.5		

For our process, wittig reaction generating Naphthacene-2,3-dicarboxylic acid diethyl ester

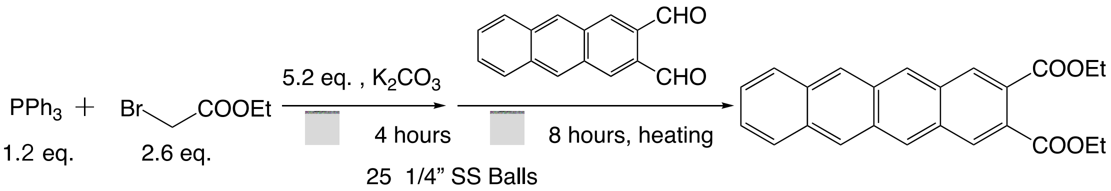

Parameter	Item	Penalty points	Note
Yield	20%	40	
Price of reaction components (to obtain 10 mmol of end product)	2,3-Naphthalenedicarboxaldehyde	5	Lab-made starting material, $942 Based on previous calculation
	Triphnyl phosphine	0	
	Ethyl bromoacetate	0	
	Potassium carbonate	0	

Safety	2,3-Naphthalenedicarboxaldehyde	N/A	Lab-made starting material, safety information not found
	Triphnyl phosphine	0	
	Ethyl bromoacetate	5	toxic
	Potassium carbonate	0	
Technical set-up	Common setup	0	
Temperature/time	Heating, > 1 h	3	
Workup and purification	Simple filtration	0	
	Classical chromatography	10	
Eco-scale point	100-63=37		

Reference

1. Lin, C.; Lin, K.; Pal, B.; Tsouz, L., *Chem. Commun.*, **2009**, 803-805
2. Angus, R.O.; Johnson, R. P., *J. Org. Chem.*, **1983**, *48*, 273-276
3. Wilcox, C. F. Jr.; Weber, K. A., *J. Org. Chem.*, **1986**, *51*, 1088-1095
4. Achar, T. K.; Maiti, S.; Mal, P. *RSC Adv.*, **2014**, *4*, 12834-12839
5. Firouzabadi, H.; Mostafavipoor, Z., *Bull. Chem. Soc. Jpm.*, **1983**, *56*, 914-917
6. Mallouli, A., Lepage, Y., *Synthesis*, **1980**, *9*, 689
7. Andersen, J.; Mack, J. *Chemical Science* **2017**, *8*, 5447-5453
8. K. Van Aken, L. Strekowski and L. Patiny, *Beilstein J. Org. Chem.*, **2006**, *2*, No. 3, No pp. given.
